# Magnetically induced convection enhances water electrolysis in microgravity

**DOI:** 10.1038/s41557-025-01890-0

**Published:** 2025-08-18

**Authors:** Ömer Akay, Macià Monfort-Castillo, Theo St Francis, Julian Becker, Shaumica Saravanabavan, Álvaro Romero-Calvo, Katharina Brinkert

**Affiliations:** 1 ZARM—Center of Applied Space Technology and Microgravity, University of Bremen, Bremen, Germany; 2 Daniel Guggenheim School of Aerospace Engineering, Georgia Institute of Technology, Atlanta, GA USA; 3 Department of Chemistry, University of Warwick, Coventry, UK

**Keywords:** Electrochemistry, Chemical engineering, Nanoscale materials

## Abstract

Since the early days of space exploration, the efficient production of oxygen and hydrogen via water electrolysis has been a central task for regenerative life-support systems. Water electrolysers are, however, challenged by the near-absence of buoyancy in microgravity, resulting in hindered gas bubble detachment from electrodes and diminished electrolysis efficiencies. Here we show that a commercial neodymium magnet enhances water electrolysis with current density improvements of up to 240% in microgravity by exploiting the magnetic polarization of the electrolyte and the magnetohydrodynamic force. We demonstrate that these interactions enhance gas bubble detachment and displacement through magnetic convection and achieve passive gas–liquid phase separation. Two model magnetoelectrolytic cells, a proton-exchange membrane electrolyser and a magnetohydrodynamic drive, were designed to leverage these forces and produce oxygen and hydrogen at near-terrestrial efficiencies in microgravity. Overall, this work highlights achievable, lightweight, low-maintenance and energy-efficient phase separation and electrolyser technologies to support future human spaceflight architectures.

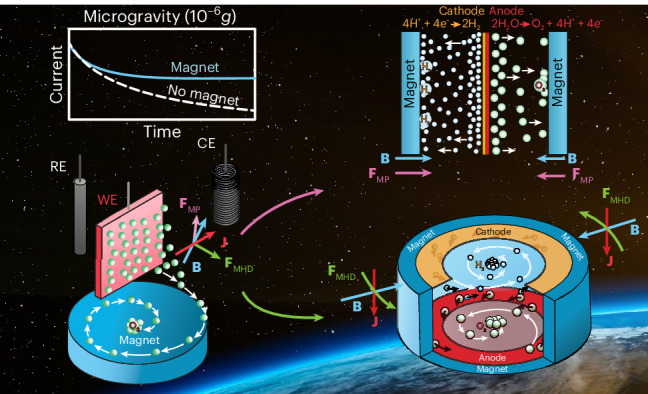

## Abstract (web summary)

Electrochemical devices producing oxygen in space face limited buoyancy, hindering gas bubble removal and increasing reaction overpotentials. Now, it has been shown that commercial magnets can enhance the reaction efficiency and induce phase separation in microgravity. The optimized magnetoelectrochemical architectures developed here may guide the designs of future life-support systems.

## Main

Since the early 1960s, the reliable and continuous supply of oxygen produced at minimal energy input has been a major obstacle faced by human space missions. The near-absence of buoyancy in orbit severely hinders phase separation and complicates the electrolysis of water on the International Space Station (ISS) and in future space exploration architectures for regenerative oxygen and hydrogen production^1–5^. Currently, electrochemical water-splitting is achieved in microgravity (10^−6^*g* or μ*g*) through substantial instrumental and energetic efforts: the proton-exchange membrane (PEM) electrolyser cell stack in the Oxygen Generator Assembly (OGA) on the ISS is connected to the Rotary Separator Accumulator (RSA), which separates the product gases from water by centrifugation^6,7^. The energetic requirements for this process and the additional overpotentials necessary to split water due to the hindered gas bubble detachment from the electrode surfaces result in a combined power consumption of ∼1.5 kW, constituting roughly a third of the energy used by the entire Environmental Control and Life Support System (ECLSS)^8^. Moreover, the complexity of the system inevitably leads to multiple failure modes and reduced reliability^6,9^, which in turn increases the up-mass cost through the addition of spare components^10^. As of today, existing oxygen generation systems do not result in mass or cost reductions compared with transporting oxygen in storage tanks on long-term space missions to Mars^11^. These limitations directly impact the mass and power budgets of the mission, and therefore its feasibility. There is thus a clear need for alternative, lightweight and reliable solutions that also offer easier maintainability.

The impact of a magnetic field on water electrolysis has been investigated for several decades in terrestrial applications^12–22^. The line of research on electrochemical processes focuses on magnetohydrodynamic (MHD) interactions originating from the Lorentz force

1 $${{\mathbf{F}}}_{{\rm{MHD}}}={\mathbf{J}}\times {\mathbf{B}}$$

that arises as a cross-product between the electric current density (**J**) and magnetic (**B**) fields. The MHD effect has been shown to increase the efficiency of electrolysis due to macro- and microscopically induced convection that improves gas bubble detachment from the electrode surface^17,23,24^. This forced convection mechanism decreases the diffusion boundary layer thickness at the electrode–electrolyte interface and increases the mass transport rate as well as the limiting current density^13^. The diamagnetic or paramagnetic polarization (MP) force^25^

2 $${{\mathbf{F}}}_{{\rm{MP}}}={\mu}_{0}M\;{\nabla}H$$

where *μ*_0_, *H* and *M* are the magnetic permeability of free space and the magnetic and magnetization field modules, respectively, produces a localized magnetic buoyancy effect due to the differential magnetic permeability between liquid and gas phases. Because *M* = *χ*^vol^*H*, with *χ*^vol^ being the magnetic susceptibility of the substance, liquids with a high magnetic susceptibility are subject to larger magnetic polarization forces. This effect can be used to direct gas bubbles in aqueous solutions to specific collection points in microgravity^26^, but not terrestrially, where the diamagnetic acceleration is at least two orders of magnitude weaker than gravity. In microgravity, the ratio between MHD and MP forces leads to the non-dimensional number

3 $${\mathscr{M}}{\mathscr{\propto }}\frac{I}{{\chi}^{\,{\rm{vol}}}{Hl}}\,$$

with *I* being the electrode current and *l* being the characteristic length. Both magnetic phase separation methods—based on different physical phenomena—apply to various water electrolysis technologies in space environments^27^. Despite holding promise to simplify phase separation and improve device efficiencies, MHD and MP forces remain largely unexplored in microgravity electrolysis. The high reliability resulting from the lack of moving components in these flow-control methods can, however, lead to mission-enabling advances in critical areas such as life support or spacecraft propulsion.

## Results and discussion

### Magnetoelectrochemical characterisation

**Fig. 1:**
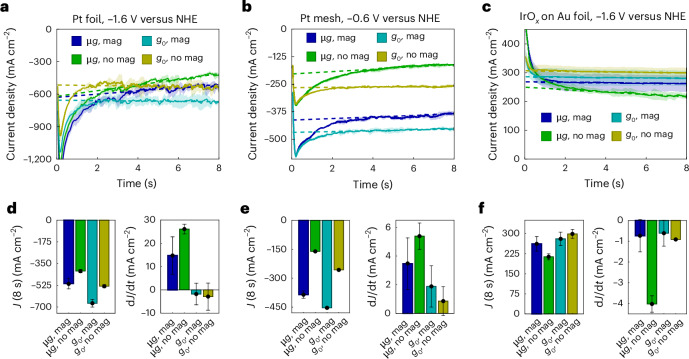
Chronoamperometric hydrogen and oxygen evolution measurements using polycrystalline platinum (HER) and IrO_*x*_ (OER) electrodes in the presence and absence of a magnetic field in terrestrial (*g*_0_) and microgravity (µ*g*) environments. **a**–**c**, Measurements were carried out in the HER (**a** and **b**) and OER (**c**) regions for 8 s each. A Pt foil electrode and a Pt mesh electrode were used in **a** and **b**, respectively. mag, magnetic field. Linear regression analyses were performed for *t* > 3 s in **a**–**c**. **d**–**f**, The slope of the current density decrease over time (d*J/*d*t*, right) and the current density at 8 s (left). The data presented in **d** correspond to **a**, panel **e** shows the data analysis for **b** and panel **f** shows the analysed data for **c**. Data are presented as mean values ± 1 s.d. from 3 independent measurements (*n* = 3). The corresponding video recordings are available as Supplementary Videos 1–3. Source data

In a series of drop tower experiments carried out at the Center of Applied Space Technology and Microgravity (ZARM, Bremen, Germany), where high-quality microgravity conditions (10^−6^*g*) can be generated for up to 9.3 s during free fall^28^, the impact of MHD and MP forces was investigated on water electrolysis. Initially, the hydrogen evolution reaction (HER) and the oxygen evolution reaction (OER) were addressed separately in a three-electrode half-cell configuration with a 1 M HClO_4_ (aq.) electrolyte solution. A polycrystalline platinum foil or mesh was used as the working electrode for the HER, whereas IrO_*x*_ deposited onto a gold substrate was used as the working electrode for the OER. Chronoamperometric measurements were carried out terrestrially and in microgravity with and without an optimally oriented N52 cylindrical permanent magnet (NdFeB, ~0.6 T) placed under the working electrode (Fig. 1 and Supplementary Fig. 1). The optimal orientation maximizes the cross-product in equation (1) by making the magnetic flux density and current flow fields perpendicular. Video recordings of all relevant experiments in microgravity and terrestrial environments are available as Supplementary Videos 1–9.

Without magnetic fields, a current density of 410.7 ± 13.8 mA cm^−2^ was measured for the HER at −1.6 V versus NHE using platinum foil electrodes (Fig. 1a). This value is nearly 30% lower than the current density measured under terrestrial conditions (532.1 ± 11.7 mA cm^−2^). In close proximity to the magnet (Supplementary Fig. 2a), the current density in microgravity increased by nearly 25% to 511.1 ± 42.8 mA cm^−2^. Terrestrially, the current density increased by 26%, yielding 669.8 ± 31.0 mA cm^−2^. The current enhancement was even stronger with a platinum mesh electrode (Fig. 1b): with a magnetic field, the overall current density increased from 256.8 ± 5.3 mA cm^−2^ to 453.8 ± 4.8 mA cm^−2^. In microgravity, the platinum mesh electrode yielded a current density of 160.7 ± 3.5 mA cm^−2^ for the HER at −0.6 versus NHE, which increased by nearly 240% to 385.3 ± 19.0 mA cm^−2^ in the presence of a magnetic field. Capillary meshes are regularly used as gas bubble barriers in microgravity due to the large differential pressure required to break the capillary interface between strings^29^. This also causes the significantly reduced HER overpotential observed here, where the small pore and strand widths^30^ combined with enhanced convection originating from the magnetic polarization of the diamagnetic electrolyte in equation (2) and the Lorentz force in equation (1) accelerate gas bubble movement. The diamagnetic liquid (HClO_4_ (aq.)) is repelled from the magnet and forces gas bubbles to move in the opposite direction, creating a magnetic buoyancy effect^26^. Because the magnetic susceptibility of oxygen (${\chi }_{{\rm{O}}_2}^{{\rm{vol}}}=3.73\times {10}^{-7}$) and hydrogen (${\chi }_{{\rm{H}}_2}^{{\rm{vol}}}=-1.00\times {10}^{-10}$) are orders of magnitude below that of water (${\chi }_{{\rm{H}}_2{\rm{O}}}^{{\rm{vol}}}=-9.1\times {10}^{-6}$), the method is largely independent of the gas composition^26,31^. Depending on the current density and the resulting ${\mathscr{M}}$ value, the MHD force can impose significantly larger liquid accelerations than the diamagnetic force (Supplementary Fig. 2b,c).

MHD- and MP-induced efficiency enhancements are also observable in the OER half-cell: Fig. 1c shows that at +1.74 V versus NHE, a current density of 213.4 ± 11.3 mA cm^−2^ was measured after 8 s of free fall with IrO_*x*_ electrodes fabricated on a gold substrate; 299.0 ± 16.4 mA cm^−2^ was achieved terrestrially. Magnetic-field-related improvements were not visible terrestrially (280.5 ± 25.1 mA cm^−2^)—potentially due to the slower reaction kinetics—but the current density improved by about 23% to 261.7 ± 27.3 mA cm^−2^ in microgravity.

Photoelectrodes used for light-assisted hydrogen production showed smaller effects in microgravity. Here, p-InP coated with a hydrophilic, nanostructured rhodium electrocatalyst particles served as an integrated semiconductor–electrocatalyst system^32,33^. At −0.09 V versus NHE and illumination with a tungsten–iodine lamp (89 mW cm^−2^), the photoelectrodes produced 24.6 ± 1.0 mA cm^−2^ without a magnetic field during the 8 s of free fall, which is in line with previously obtained results. Due to the low current density—producing a small Lorentz force—and reduced bubble coverage, only slight improvements were recorded in the presence of a magnetic field, yielding 25.6 ± 1.6 mA cm^−2^ (Supplementary Fig. 3 and Supplementary Video 4). The same trend was observed with a platinum foil electrode during the HER at low current densities: without a magnet at −0.1 V versus NHE, a current density of 24.1 ± 2.8 mA cm^−2^ was measured at 8 s. The current density slightly improved to 25.0 ± 2.5 mA cm^−2^ with a magnet (Supplementary Fig. 4 and Supplementary Video 5). In both cases, the diamagnetic properties of the electrolyte are the major contribution to the movement of hydrogen gas bubbles from the electrode surface following the magnetic field gradients. The impact on the electrochemical performance only becomes observable towards the end of the free-fall period. Video recordings of both electrochemical systems (Supplementary Videos 4 and 5) show, however, evidence of the induced gas bubble movement in microgravity in the presence of a magnetic field.

Due to the absence of mechanical electrolyte stirring, a current density decay was observed in all experiments (shown as d*J*/d*t* in Fig. 1). With a magnetic field, however, the decay was significantly slowed down due to the enhanced convection. This was particularly noticeable for the platinum foil and IrO_*x*_ experiments (Fig. 1d,f), in which hydrogen and oxygen gas were produced at higher current densities. The current density decay in microgravity decreased in the presence of a magnetic field from 26.1 ± 2.1 mA cm^−1^ s^−1^ to 14.8 ± 8.0 mA cm^−1^ s^−1^ for the HER and from 4.0 ± 0.4 mA cm^−1^ s^−1^ to 0.7 ± 0.8 mA cm^−1^ s^−1^ for the OER. The magnetic field impact is expected to outcompete the effects of a mechanical stirrer particularly in longer-duration microgravity experiments because additional vortexes at the electrode edges further enhance gas bubble detachment (Supplementary Video 6 and Supplementary Figs. 5 and 6).

### Gas bubble evolution dynamics

**Fig. 2:**
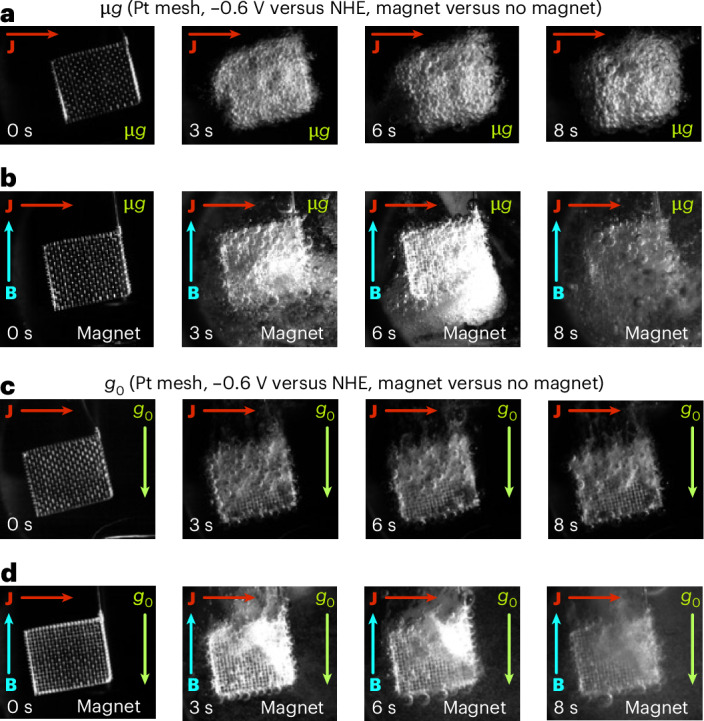
Time series of hydrogen gas bubble evolution during chronoamperometric measurements on platinum mesh electrodes in the presence and absence of a magnetic field in *g*_0_ and µ*g.* **a**, Without a magnetic field, hydrogen gas bubbles coalesce and form a froth layer on a polycrystalline platinum mesh electrode in microgravity. **b**, The magnet at the bottom of the platinum electrode attracts hydrogen gas bubbles through magnetic polarization of the electrolyte and induces a convective flow through MHD stirring. **c**, For comparison, terrestrial HER experiments were carried out with the same electrode. **d**, Enhanced convection and gas bubble removal occurred in the presence of a magnetic field due to the magnetic forces. In all cases, the potential was set to −0.6 V versus NHE; 1 M HClO_4_ (aq.) with the addition of 1% (v/v) isopropanol was used as the electrolyte. All video recordings are available as Supplementary Video 2.

Although the electrochemical characterization informs the efficiency of the system, the analysis of gas bubble dynamics is the basis for the development of microgravity phase separators. Clear differences between non-magnetic and magnetic cells were observed during the drop tower experiments. Figure 2 compares hydrogen bubble evolution over the platinum mesh electrode from Fig. 1b without (Fig. 2a) and with (Fig. 2b) magnetic forces in microgravity and shows the equivalent terrestrial experiments for comparison (Fig. 2c,d). While a static foam layer was formed on the non-magnetic electrode in microgravity, the behaviour radically changed when the combination of MHD and MP effects induced by the permanent neodymium magnet started to stir the flow at −0.6 V versus NHE. Small hydrogen bubbles were generated and quickly detached from the surface of the platinum mesh, initiating an MHD-induced spiralling motion towards the magnet under the influence of the MP force. As the experiment progressed, the number of bubbles increased, but the initial flow pattern remained stable without regressing gas bubble transport. Qualitatively, this pattern was similar to that observed in terrestrial experiments without and with a magnet (Fig. 2c,d, respectively). Foam did not accumulate at the meshes and, instead, the bubbles vigorously moved upwards due to gravity. MHD forces resulting from the current flow and the geometry of the cell were, however, sufficient to cause a vortex motion of the electrolyte. Similar observations were made with platinum foil and IrO_*x*_ electrodes for the HER and OER (Supplementary Figs. 7 and 8).

Following the magnetic acceleration maps computed in Supplementary Fig. 2b,c, the diamagnetic electrolyte polarization drives hydrogen bubbles towards the magnet with liquid accelerations of 10–100 mm s^−2^. This force is determined by the position of the bubble relative to the magnet and is independent of the liquid–gas distribution, because the magnetic susceptibility of the liquid–gas mixture is too small to induce significant changes in the local magnetic field^26^. The Lorentz force generates an average horizontal acceleration of 50–250 mm s^−2^ at a current density of ~385 mA cm^−2^ as sketched in Supplementary Fig. 1b. Due to the small volume of the test cell in Supplementary Fig. 1a and the offset of the working electrode and counter-electrode from the axis of symmetry of the liquid volume, a net torque was applied to the liquid, producing the rotational pattern. Additionally, the Lorentz force distribution depends on the local magnetic and electric current density field directions. The presence of gas bubbles modifies these current density lines^34^ and induces second-order flow disturbances.

Because the diamagnetic force only depends on the magnetic susceptibility of the electrolyte and the magnetic field distribution described in Supplementary Fig. 2b, the current density field sets the strength of the Lorentz force on the liquid and determines the relative importance of both effects. Following equation (3), three distinct flow regimes are observed depending on the current density value: (1) a diamagnetically dominated case with ${\mathscr{M}}{\mathscr{\ll }}1$ in which bubble trajectories follow the magnetic field lines^26,27^; (2) a forced MHD regime with ${\mathscr{M}}{\mathscr{\gg }}1$ in which bubbles advect with the liquid; and (3) a mixed state in which both effects drive the gas bubble behaviour (${\mathscr{M}}{\mathscr{\approx }}1$). The platinum mesh experiment depicted in Fig. 2b with ${\mathscr{M}}{\mathscr{\approx }}4.33$ is an example of the MHD regime, while the photoelectrode test in Supplementary Video 4 with ${\mathscr{M}}{\mathscr{\approx }}0.84$ exemplifies the mixed scenario.

### Magnetoelectrochemical architectures for oxygen production

To reliably and efficiently generate oxygen, any life-support architecture for human spaceflight must tackle the fundamental obstacle of gas separation. The OGA on the ISS and its upcoming iterations implement a water recirculation loop with pumps, centrifuges and a complex flow-control process^1,2^. As with any other space system, the presence of moving parts leads to multiple failure modes (for example, a pump lock-up^35^) that—in the case of the OGA—make it unsuitable for the transit to Mars^10^. The touchless diamagnetic and MHD flow-control strategies explored here offer a solution to this problem.

#### Diamagnetically driven proton-exchange membrane electrolysis

Due to their high current density and inherent operational safety caused by the physical separation of the hydrogen and oxygen chambers, catalyst-coated PEMs tend to be the preferred water electrolysis architecture in space. In PEM cells, ions travel through a solid membrane rather than the surrounding electrolyte, eliminating current flow in the liquid. This restricts magnetic forces to MP effects only, enabling simplified bubble separation architectures.

**Fig. 3:**
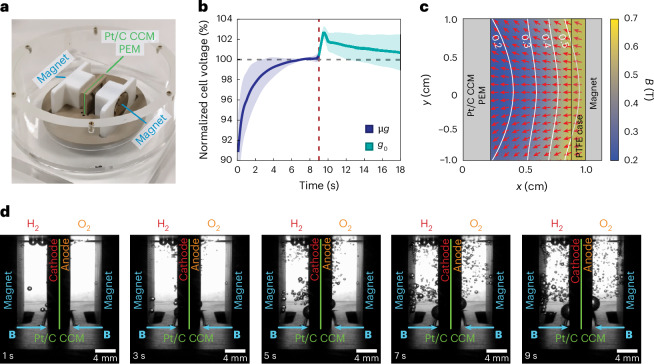
Diamagnetically driven PEM water electrolysis in microgravity. **a**, Photographic image of the PEM electrolyser model cell set-up used. **b**, Normalized time-dependent cell voltage measurement in the model PEM cell at a constant current of 200 mA, firstly recorded in microgravity and directly afterwards, terrestrially. The dashed red line after 9.2 s represents the deceleration of the cell. Data are presented as normalized mean values ± 1 s.d. from 3 independent measurements (*n* = 3). The raw data of the full experiment are shown in Supplementary Fig. 10. **c**, Magnetic field simulation between the magnet and the PEM. *x* and *y* denote the horizontal and vertical coordinates in the symmetry plane of the PEM cell, respectively. **d**, Time series of hydrogen and oxygen gas bubble evolution in chronopotentiometric measurements in the PEM electrolyser model cell in the presence of magnetic fields. Photographs were taken at 1 s, 3 s, 5 s, 7 s and 9 s during free fall. The magnetic repulsion from the permanent magnets (highlighted in blue) positioned on either side of the catalyst-coated PEM (CCM, highlighted in green) effectively directs gas bubbles towards the magnets, facilitating efficient phase separation. The experiment was carried out with ultrapure water (18.2 MΩ cm) as the electrolyte. The video recording is available as Supplementary Video 7. Source data

A model PEM cell was constructed to demonstrate the MP phase-separation effect. A platinum-coated PEM pressed between platinum meshes was utilized on both the anodic and cathodic sites. The mesh electrodes were then pressed between polyether ether ketone (PEEK) frames and connected with platinum wires. The set-up was encapsulated in a PEEK electrolyser chamber with an acrylic glass surround for visual observation (Fig. 3a and Supplementary Fig. 9). Chronoamperometric measurements at 200 mA were carried out in ultrapure water (18.2 MΩ cm) in microgravity and directly afterwards in terrestrial environments with the same duration. It was observed that the cell voltage increased during the first second and began to saturate after 3–4 s (Fig. 3b). The deceleration shock caused a quick voltage rise that decreased in the following seconds to approximately the values achieved in microgravity due to the sudden release of gas bubbles. The feasibility of diamagnetic gas extraction from the cell with permanent magnets on each electrode site was also determined. Figure 3d shows that magnetic polarization effectively controls the dynamics of gas bubbles, facilitating their separation by overcoming the lack of buoyancy forces that assist gas bubble dynamics on Earth. This model PEM cell demonstrates that (1) phase separation induced by magnetic polarization in microgravity delivers an electrolyser performance comparable to terrestrial conditions with the ability to produce foam-free oxygen and hydrogen, and (2) diamagnetic forces can be effectively used to induce gas bubble detachment and collection (Supplementary Video 7), improving the operational stability and efficiency of PEM electrolysers in microgravity conditions. Engineering implementations of the concept will feature a gas outlet located at the surface of the magnet^27^, where gas bubbles coalesce due to the strong diamagnetic force (Supplementary Fig. 11b). As demonstrated previously^26^, the diamagnetic force is sufficient to induce bubble coalescence in microgravity. This feature—combined with the agreement between bubble velocity predictions (Methods) and experiments—lays the foundation for future engineering designs in which PEM cells are operated without moving parts in microgravity.

#### MHD drive

**Fig. 4:**
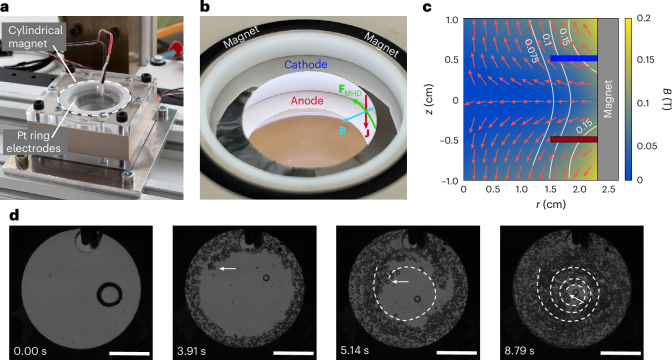
Scheme of a proof-of-concept cylindrical MHD drive electrolytic cell architecture exploiting the Lorentz force to induce vortical phase separation in microgravity. **a**, Photograph of a prototype MHD drive with platinum foil ring electrodes and a circular, segmented N52 neodymium arc magnet for efficient oxygen and hydrogen gas collection in microgravity. **b**, Close-up image of the electrode and magnet arrangement in the device. **c**, The combination of a radial magnetic field and the electrical current density between anode and cathode generates a Lorentz force on the liquid that induces a vortical flow. Phase separation is initiated due to the centrifugal acceleration and the density difference between liquid and gas phases. *r* and *z* denote the radial and vertical coordinates in the axisymmetric plane of the MHD drive, respectively. **d**, A proof-of-concept test in microgravity generated at the Bremen Drop Tower shows the expected behaviour of the MHD drive: the trajectory of a gas bubble is highlighted, providing a visual demonstration of the vortical flow and centripetal phase-separation effect (Supplementary Video 8). The current density was set to 105 mA cm^−2^ and 1 M HClO_4_ (aq.) was used as the electrolyte. Scale bars, 1 cm.

The MP force achieves gas separation by directing gas bubbles towards desired collection points, but it is bounded by the weak diamagnetic susceptibility of most liquids^26,27^. Ultimately, this limits the maximum gas flow extracted from the electrolytic cell, constraining its applicability to low-volume-flow-rate architectures. The forced MHD regime, on the contrary, induces much stronger forces on the liquid–gas mixture, but does not directly act on the bubbles and, therefore, does not by itself lead to their separation. The cylindrical architecture conceptualized in Supplementary Fig. 12 and partially realized in Fig. 4a,b solves the problem of gas bubble collection by exploiting the MHD effect as a proxy, rather than a primary phase-separation mechanism. The Lorentz force is applied circumferentially on the conducting fluid to spin the liquid–gas mixture (Supplementary Fig. 12). This generates a vortical flow that induces a centripetal force on the liquid, driving the gas to central gas outlets while pushing the liquid to the exterior wall with the resulting centripetal force. Gas bubbles coalesce in the core of the drive and can then be extracted through an outlet located at the centre using the pressure difference between the device and downstream systems. Gas recombination is prevented by placing a porous membrane between the pair of ring electrodes. Ultimately, this MHD cell architecture should generate, separate and collect hydrogen and oxygen bubbles without moving parts in microgravity.

A small-scale PEEK MHD cell prototype was developed to test the hypotheses with two circular platinum foil electrodes in a cylindrical casing (Fig. 4a,b), even though the short duration of the microgravity window prevented a demonstration of the bubble coalescence process at the core. A radially magnetized neodymium magnet comprising eight arc segments enclosed the electrode assembly and produced the magnetic field described in Fig. 4c. Figure 4d depicts the vortical separation effect during an 8 s microgravity experiment at 105 mA cm^−2^, showing the radial bubble displacement induced by the rotation of the electrolyte. Based on the work carried out by the MHD torque, the power required to spin the liquid is estimated to be ~0.1 mW in this case. Even though power conversion losses should be added to this first-order estimate, they are still negligible in comparison to the ~2.7 W required to run the electrochemical reaction. Moreover, Supplementary Fig. 13 shows that removing the magnets from the MHD drive results in a 12% increase in power consumption, presumably due to the increased bubble coverage in the absence of MHD stirring. This highlights the low power requirements of the MHD phase separation method and its effectiveness for gas bubble removal.

**Fig. 5:**
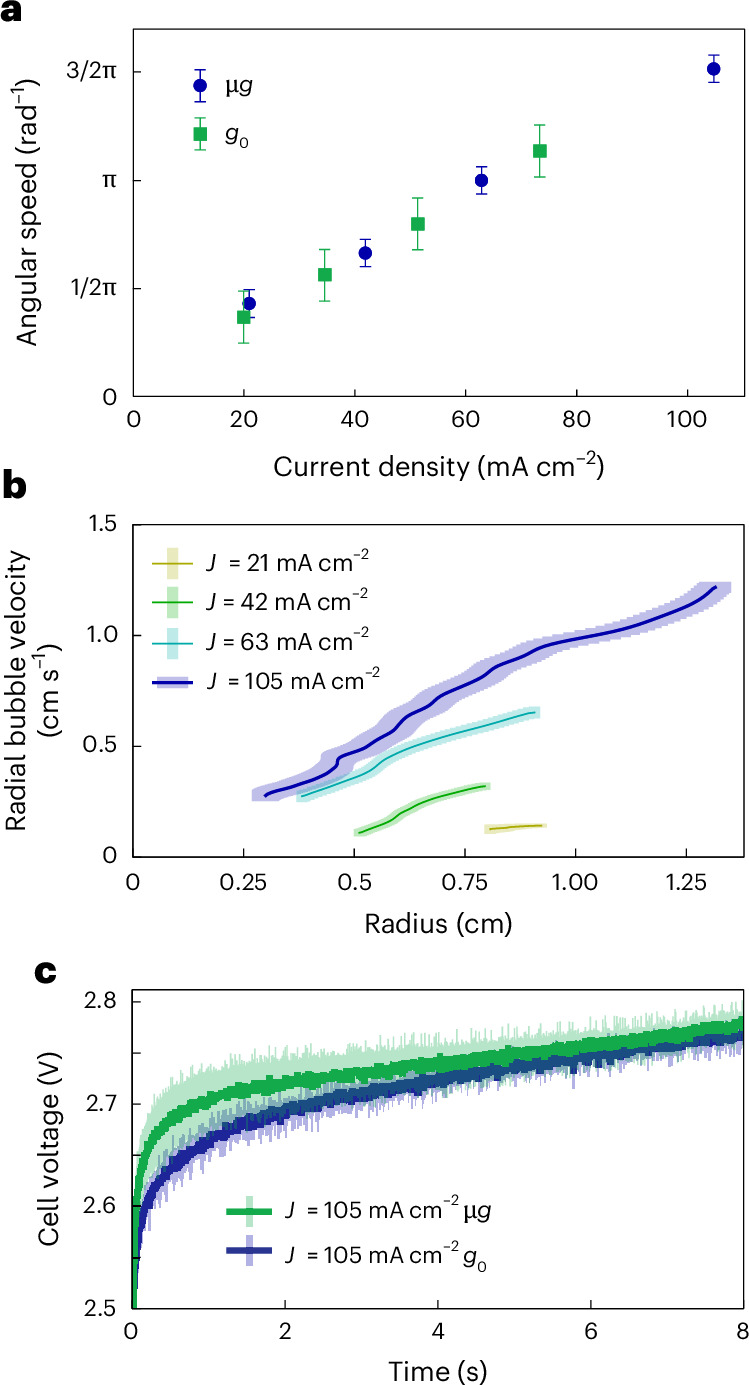
Performance measurements of the MHD drive in microgravity and terrestrial environments. **a**, Variation of the angular velocity of the electrolyte with current density for both microgravity and terrestrial environments. Data are presented as mean values ± 2 s.d. of three independent measurements. **b**, Variation of the radial velocity of gas bubbles that showed steady-state behaviour (Methods) for different current densities and radial positions. The error bars represent the difference between the fit used for the radial position derivative (Methods) and the actual radial position ±2 s.d. **c**, Chronopotentiometric measurements of the MHD drive at 105 mA cm^−2^ indicate a very similar operation and efficiency in both terrestrial and microgravity environments. Data are presented as mean values ± 2 s.d. of three independent measurements. Source data

The angular velocity of the electrolyte within the MHD drive determines how strongly the liquid is accelerated towards the lateral walls and how fast the gas bubbles reach its centre. Values between 15/36π and 55/36π rad s^−1^ were measured from video footage using gas bubbles as visual markers for current densities between 21 and 105 mA cm^−2^ (Supplementary Video 8). These angular velocity values are approximately uniform across all radii, indicating that the liquid rotates like a rigid body. Figure 5a shows the angular bubble velocity as a function of the current density for terrestrial and microgravity experiments. A linear trend was observed in both cases, showing that the same governing physical effects are present. The radial bubble velocity of representative bubbles was plotted as a function of the radius and for different current densities in Fig. 5b (see also Supplementary Fig. 14), with values ranging from 0 to 1.25 cm s^−1^. Because the radial bubble velocity depends on the centripetal acceleration, smaller linear velocities were observed near the centre.

For the electrochemical characterization of the MHD drive, a constant current of 1,000 mA was applied between the platinum anode and cathode, resulting in a total current density of 105 mA cm^−2^. The chronopotentiometric characteristics of the device were recorded terrestrially and during free fall (Fig. 5c). The MHD drive showed very similar electrochemical characteristics in microgravity and terrestrial environments, demonstrating the possibility to construct a system that maintains constant bubble formation with statistically the same efficiency as observed in terrestrial measurements (Supplementary Video 9).

## Conclusions

Reliability, mass and power are the driving factors for the design of life-support systems for space exploration, particularly for oxygen production. We have shown that commercial off-the-shelf magnets can be utilized to operate electrolytic water-splitting cells for hydrogen and oxygen production in microgravity at near-terrestrial efficiencies. The diamagnetic and Lorentz forces originating from the magnetic polarization of the electrolyte and the interaction between magnetic and current density fields, respectively, could promote gas bubble detachment and movement in microgravity to the extent that current density improvements of up to 240% were recorded for the HER with a platinum mesh working electrode in a three-electrode set-up. To exploit these two forces for electrolytic water-splitting in microgravity, two devices were designed: one model PEM electrolyser cell that utilizes the diamagnetic force for efficient oxygen and hydrogen gas collection; and an MHD drive cell that leverages the geometric properties of the MHD force by inducing a rotational movement in the fluid that leads to vortical gas–liquid phase separation. In microgravity, both devices demonstrated operation at near-terrestrial electrochemical efficiencies while featuring a simplified design that replaces mechanical flow-control mechanisms and moving parts. The demonstrators provide a proof-of-concept for the utilization of magnetically induced flow control as a lightweight, energy-efficient and reliable phase-separation approach in electrolytic cells that pave the way for the development of next-generation electrolytic water-splitting devices for application in space environments.

## Methods

### General notes

All aqueous solutions were prepared with ultrapure water (18.2 MΩ cm, Veolia). Chemicals were purchased with an analytical grade containing <50 ppb of impurities. All electrochemical potentials are converted in the following to those versus the potential of the normal hydrogen electrode (NHE) if not indicated otherwise. Throughout the paper, the magnetic susceptibility of the electrolyte is assumed to be comparable to that of ultrapure water for first-order diamagnetic force estimation.

### Fabrication of platinum foil electrodes

Polycrystalline platinum foil, measuring 50 mm × 50 mm with a thickness of 0.1 mm, was obtained from Goodfellow Cambridge with a purity grade exceeding 99.99+% and a polished surface. For electrode fabrication, the platinum sheets were cut into 10 mm × 10 mm pieces. A copper wire with a purity level exceeding 99.999% (Thermo Scientific) and a diameter of 0.127 mm was attached to the backside of one platinum piece using conductive silver epoxy adhesive (8331D, MG Chemicals). Electrodes were then cured for 2 h at 70 °C on hotplates, ensuring a stable electrical connection.

Subsequently, each platinum electrode was threaded through a Schott Duran glass tube (Gaßner Glastechnik), measuring 8 cm in length, 5 mm in diameter and 0.8 mm in thickness. The backside of the electrode was then encapsulated with a chemically resistant, two-component polyurethane resin (UR 5528, Electrolube). The epoxy was cured for 12 h at 90 °C.

### Fabrication of platinum mesh electrodes

Platinum mesh (gauze) electrodes were obtained from ALS Japan (011498 SEC-C platinum gauze working electrode, 99.99% purity) with a total width of 7 mm, a height of 6 mm and a wire diameter of 0.08 mm. A copper wire was clamped to the end of the platinum wire to establish an electrical contact. The electrode was then threaded through a Schott Duran glass tube (Gaßner Glastechnik) with the same dimensions as above. To protect the connection between the platinum and copper wire, it was encapsulated with polytetrafluoroethylene (PTFE) tape. This tape also provided stability and orientation of the electrode. Hot glue sealed the end of the glass tube.

### IrO_*x*_ electrode fabrication

IrO_*x*_ was electrodeposited onto gold electrodes following an adaptation of a previously published protocol^36^.

#### Gold electrode preparation

Gold foil (99.95%, 0.1 mm thick, Strem Chemicals) was cut into 10 mm × 10 mm pieces, sonicated for 5 min in ethanol and for 5 min in water, and then dried in a nitrogen stream (99.999% purity). Copper wire (≥99.9%, 1.0 mm diameter, Sigma-Aldrich) was glued to the back of the gold pieces with electrically conductive silver epoxy adhesive. Copper wire was threaded through a Schott Duran glass tube. Electrode back, edges and corners were covered with epoxy resin (ER2218, Electrolube). The gold electrodes were voltammetrically cycled in 0.5 M H_2_SO_4_ (aq.) (potential range, −0.21 V to +1.49 V versus Ag/AgCl, 3 M KCl (Dri-Ref 5, World Precision Instruments); counter-electrode, platinum coil (A-002234, BioLogic); scan rate, 100 mV s^−1^; cycles, 80), rinsed with water, dried in a nitrogen stream and stored in vacuum prior to IrO_*x*_ deposition.

IrO_*x*_ was deposited by chronoamperometry in an electrodeposition bath (at +0.6 V versus Ag/AgCl; counter-electrode, platinum coil (A-002234, BioLogic); duration, 1.15 h). The electrodeposition bath was prepared by dissolving 0.15 g iridium(IV) chloride monohydrate (IrCl_4_·H_2_O, ≥99.9%, Sigma-Aldrich) in 100 ml water and the solution was stirred for 30 min. Then, 1 ml hydrogen peroxide solution (H_2_O_2_:H_2_O, 30% (w/w), Sigma-Aldrich) was added and the solution was stirred for another 10 min. Finally, 0.5 g oxalic acid dihydrate ((COOH)_2_·2H_2_O, ≥99%, Sigma-Aldrich) was added, followed by another 10 min of stirring. The solution pH was adjusted to 10.5 by incremental addition of anhydrous potassium carbonate (K_2_CO_3_, ≥99%, Sigma-Aldrich). The solution was stirred for 3 days before IrO_*x*_ deposition.

### Electrochemically active surface area determination

The hydrogen underpotential deposition method was used to determine the electrochemically active surface area (ECSA) of the platinum mesh electrodes^37^. Electrodes were cleaned with piranha solution (H_2_SO_4_:H_2_O_2_, 3:1). Subsequent electrochemical surface cleaning was performed in 0.1 M HClO_4_ (aq.) solution saturated with argon (99.999% purity) using CV between +0.05 V to +1.1 V versus NHE at a scan rate of 50 mV s^−1^ for 50 cycles. A platinum coil served as the counter-electrode with an Ag/AgCl (3 M KCl, Dri-Ref 5, World Precision Instruments) reference electrode. Afterwards, the electrolyte was exchanged, saturated again with argon and another CV was recorded under identical conditions for ECSA evaluation. After subtracting the double-layer currents, the hydrogen underpotential deposition region (+0.03 V and +0.34 V versus NHE) was integrated in both anodic and cathodic scan directions, and averaged. The coulombic charge associated with the adsorption and desorption of submonolayers of hydrogen atoms on the mesh surface was converted to the ECSA by assuming a specific charge of polycrystalline platinum of 176 µC cm^−2^, resulting in a surface area of approximately 0.87 cm^2^.

The geometric areas of the IrO_*x*_ and platinum foil electrodes were measured in triplicate from photographic images on millimetre paper using the ImageJ software (v.1.54g).

### Fabrication of p-type InP-Rh photoelectrodes

Single-crystalline p-InP wafers (111 A orientation) were purchased from AXT-Tongmei with a zinc doping concentration of 5 × 10^17^ m^−3^. To fabricate an ohmic back contact, 4 nm gold, 80 nm zinc and 150 nm gold were evaporated on the backside of the wafer and tempered at 400 °C for 60 s. The 1-cm^2^ polished indium surface of p-InP was etched in a bromine/methanol solution (0.05% w/v) for 30 s, rinsed with ethanol and ultrapure water (18.2 MΩ cm, Veolia), and dried under a nitrogen flow. Furthermore, the p-InP surface was photoelectrochemically conditioned in 0.5 M HCl (aq.) by potentiodynamic cycling in a three-electrode set-up under illumination (100 mW cm^−2^) between −0.44 V and +0.31 V versus NHE at a scan rate of 50 mV s^−1^ while purging with argon (99.999% purity). A platinum coil (BioLogic, A-002234) was used as the counter-electrode and an Ag/AgCl (3 M KCl, Dri-Ref 5, World Precision Instruments) served as the reference electrode. Illumination was carried out through a quartz window of the borosilicate glass cell with an xenon arc lamp (300 W, Newport Spectra-Physics) using an AM 1.5 G filter. The light intensity of 100 mW cm^−2^ was adjusted with a calibrated silicon reference photodiode.

Shadow nanosphere lithography was used to produce hydrophilic rhodium nanostructures on the p-InP substrate. To create the required masks for the subsequent catalyst deposition process, monodisperse polystyrene beads with a diameter of 782 nm (microparticles GmbH) were used as supplied in their aqueous suspension at a concentration of 5% (w/v). For the final solution of 600 μl, 300 μl of the polystyrene bead dispersion was mixed with 300 μl of ethanol containing 1% (w/v) styrene and 0.1% sulfuric acid (v/v). The solution was applied to an air–water interface using a Pasteur pipette with a self-made curved tip. To increase the area of the single-crystalline structures, the Petri dish was carefully moved to create weak wave motions, transforming several smaller particle domains into larger ones. The solution was spread to cover ~80% of the water surface with a hexagonally closed-packed monolayer, leaving space for stress relaxation and avoiding the formation of cracks in the lattice during the subsequent preparation steps. Photoelectrochemically conditioned p-InP electrodes were placed under the floating, closed-packed polystyrene bead mask in the Petri dish. The remaining water was carefully removed by pumping and evaporation while the mask was deposited onto the electrode. After the surface had been dried with argon, rhodium was photoelectrochemically deposited through the polystyrene particles from an aqueous solution of 5 mM RhCl_3_, 0.5 M NaCl and 0.5 vol% 2-propanol for 5 s at a constant potential of V_dep_ = + 0.01 V versus NHE and a light intensity of 100 mW cm^−2^, using the same electrode configuration and illumination source as for the photoelectrochemical conditioning. The electrodeposition resulted in the formation of a nanostructured surface morphology that resembles a honeycomb structure caused by the inverse image of the overlying mask.

Polystyrene beads were removed from the surface by placing the electrodes for 20 min in a beaker of toluene with a magnetic stir bar. The electrodes were cleaned by rinsing with acetone and ethanol for 20 s each. To remove residual carbon traces, argon plasma cleaning (MiniFlecto, Plasma Technology) was carried out at 0.16 mbar, 65 W and a gas inflow of 1 sccm for 5 min.

### Electrolyte preparation

Alongside previous HER experiments in microgravity, a 1 M HClO_4_ (aq.) solution was used as the electrolyte for all HER experiments with the addition of 1% (v/v) 2-propanol to reduce the surface tension and facilitate enhanced gas bubble detachment from the electrode^32,33^. For the OER, a 1 M HClO_4_ (aq.) solution was used as the electrolyte without any further additions. Freshly prepared electrolyte solutions were used for all terrestrial and microgravity experiments.

### Electrochemical cleaning of platinum electrodes

Prior to each experiment, platinum electrodes underwent a thorough cleaning process to ensure optimal and reproducible electrochemical performance. The electrodes were initially rinsed with acetone, 2-propanol and ultrapure water for 10 s each and dried in an argon gas flow. Before the electrochemical measurement in the drop tower or terrestrially, the platinum surface was cleaned in the electrolyte used for the subsequent CV experiment between −0.5 V and +2 V versus Ag/AgCl (3 M KCl). Twenty cycles were run at a scan rate of 50 mV s^−1^ in a three-electrode set-up. A platinum coil served as the counter-electrode.

### Microgravity facility

A microgravity environment was established at the Bremen Drop Tower at ZARM. The experimental set-up was installed in a drop capsule which was shot up ~120 m to the top of the tower by a hydraulically controlled pneumatic piston-cylinder catapult system before falling into a deceleration container containing millimetre-sized hard foam polystyrene beads. The total free-fall time was up to 9.3 s. During free fall, the minimum *g*-value was about 10^−6^*g*. Electrochemical data were stored on a Matrox 4Sight GPm integrated PC unit in the drop capsule. The drop capsule was also equipped with sensors to monitor acceleration, rotation, atmospheric pressure and temperature, and had a battery power supply. The drop sequence for all electrochemical measurements was automated and started prior to each drop, waiting for triggers at launch. The drop sequence was designed to start cameras and electrochemical measurements directly after immersing the working electrode in the electrolyte using a pneumatic system in time to reach microgravity conditions. This allowed electrochemical measurements to be carried out during free fall only. After the capsule was lifted from the deceleration container, the samples were retrieved from the experimental set-up, rinsed with ultrapure water and dried with argon.

### Baseline set-ups

#### Three-electrode cell arrangement

Electrochemical experiments were carried out in the drop tower and terrestrially in a custom-made, two-compartment electrochemical cell (filling volume, 250 ml) made of PEEK (Supplementary Fig. 1). Each cell consisted of two optical windows made of quartz glass (Suprasil 1, Aachener Quarzglas; diameter, 25 mm; thickness, 3 mm) through which the front and side of the working electrode surface could be observed through optical mirrors. Each cell could perform an electrochemical measurement independently. All experiments were carried out in a three-electrode arrangement with a platinum coil counter-electrode and an Ag/AgCl reference electrode under ambient pressure. All electrodes were separated by about 1 cm. Four cameras were installed to capture gas bubble evolution and movement inside each compartment. Two monochromatic near-infrared cameras (acA1300-60gm, Basler; Cams 3 and 4, Supplementary Fig. 1) with a resolution of 1,280 × 1,024 pixels at a capture rate of 60 frames per second were attached to each cell via optical mirrors equipped with Telecentric High-Resolution lenses (WD110 series, MML1-HR110). Two Photron MC-2 Fastcam high-speed cameras (Cams 1 and 2, Supplementary Fig. 1) equipped with 35-mm Kowa LM35HC 1-inch sensor F1.4 C-mount objectives were mounted in front of each compartment. The cameras operated at 500 frames per second with a resolution of 512 × 512 pixels. Cams 3 and 4 were installed on the sides of the cell, while the high-speed cameras (Cams 1 and 2) were positioned at the front. For the analysis of gas bubble movement, the front-facing cameras were used. Optical mirrors were used to adjust the camera heights, reducing space requirements and torque during rapid acceleration. This set-up made it possible to record a static view of the gas bubble evolution and the movement trajectories of the bubbles during the experimental sequence.

Tungsten–iodine lamps provided background illumination for the video recordings from the sides (Light, Supplementary Fig. 1). When electrochemical measurements with photoelectrodes were carried out, the tungsten–iodine lamps were used for sample illumination from the front at 89 mW cm^−2^, calibrated using a silicon photodiode.

For electrochemical experiments in the presence of a magnetic field, a cylindrical N52 neodymium magnet (NdFeB, 19.05 mm length, 25.4 mm diameter, K&J Magnetics) was placed ~4 mm below the working electrode (Supplementary Fig. 1). A protective sleeve was fabricated from acidic-resistant polyoxymethylene to shield the neodymium magnet from the electrolyte. To secure the sleeves and magnets at the bottom of the cell during rapid capsule acceleration (up to 40*g*), a cylinder of the same polyoxymethylene material was constructed to precisely fit the magnet at the bottom of each compartment.

All measurements were carried out in the absence of electrolyte stirring if not indicated otherwise and were repeated three times for statistical evaluation. Analysis focused on the time period of 8 s after the onset of microgravitation to exclude differences in the deceleration onset of the drop tower capsule. The chosen time interval falls within the minimum microgravity period by a significant margin, thus ensuring the consistency and accuracy of the measurements. Terrestrial comparison experiments were carried out in the same set-up using the same experimental conditions.

#### Magnetic environment

MP and MHD forces were induced by the axially magnetized, cylindrical N52 neodymium magnet. The magnetic field generated by the magnet was computed as the superposition of the individual magnetic fields induced by *N* = 40 equivalent circular loops located at the side wall of the cylinder, which features discontinuous tangent residual magnetization components. Each loop has a current *I*_loop_ = *M*_r_*h*_magnet_/*N*, with *M*_r_ being the residual magnetization of the magnet and *h*_magnet_ its height, resulting in an analogous magnetic system with an analytical solution. Further details on the virtual currents method can be found in ref. ^38^.

Supplementary Fig. 2 characterizes the magnetic environment of the system in the plane defined by the working electrode and the counter-electrode. The magnetic flux density distribution, shown in Supplementary Fig. 2a, reaches ~0.6 T over the surface of the magnet. The diamagnetic bubble terminal velocity is depicted in Supplementary Fig. 2d and ranges between 0.1 and 2 mm s^−1^ over the surface of the electrode for a 0.5-mm-radius bubble. Diamagnetic accelerations between 10 and 100 mm s^−2^ act on the electrolyte at the same locations, but the Lorentz acceleration can be significantly larger depending on the current density value. For instance, at a current density of 200 mA cm^−2^, the average acceleration from the Lorentz force is approximately 0.5 m s^−2^, one order of magnitude higher than the diamagnetic effect. Since the mean electric current density vector **J** is contained in the plane of the electrode, the Lorentz force pushes the liquid in the circumferential direction.

#### Mechanical stirring

To investigate the impact of improved convection with a magnet in microgravity further, a mechanical stirrer was added to the electrochemical cell without a magnet (Supplementary Fig. 5). Supplementary Fig. 6 shows that mechanical stirring decreases the current density decay in microgravity comparably, supporting the hypothesis that by improving gas bubble detachment and movement from the electrode surface, the magnetically induced convection prevents the formation of a gas bubble froth layer and the blocking of catalytically active sites on the platinum electrode. This, in turn, it enhances proton interaction with the electrode surface and improves the hydrogen evolution rate.

For the mechanical stirring experiments, a motor (RH158-12-15 d.c. motor, MicroMotors; 39.6 mm, with a 15:1 gearbox, 12 V, 100 N mm) was attached to a rod, circumventing the magnetic field generated by commercially available magnetic stirrers (Supplementary Fig. 5). The rod was 120 mm in length and 6 mm in diameter. The end of the rod was attached to a thin plate, 1 mm in thickness, 30 mm in width and 10 mm in height, acting as a paddle to stir the solution. The paddle was attached to the rod by snapping it into sawed-off slots. The entire stirring rod was attached to the axis of the motor’s gearbox using grub screws. The stirring rod was placed 1.5 cm off-centre from where the working electrode was positioned. The paddles rotated below the working electrode. The voltages in Supplementary Fig. 6 were chosen in all scenarios to result in matching current densities. An Ag/AgCl electrode (3 M KCl, Dri-Ref 5, World Precision Instruments) was used as the reference and a platinum coil as the counter-electrode (A-002234, BioLogic) for all experiments.

It should be noted that during stirring, the solution was pushed towards the outer walls of the cell, forming a vortex in the centre. To maintain the pressure at 1 atm, it was necessary to keep the gas volume as low as possible. When producing hydrogen gas through the HER, the electrochemical cell was filled with 175 ml of 1 M HClO_4_ (aq.) + 1% isopropanol, leaving 20 ml unfilled. The stirring rod’s volume, including the three electrodes, occupied an additional 5 ml. The total gas volume for the stirring experiments was thus 15 ml.

Because the solution was in constant rotary motion—pushing the liquid to the outer walls—the resulting gas bubbles formed an elongated shape. Due to the off-centre placement of the stirrer and the position of the other electrodes in solution, the electrolyte flow became turbulent, causing the bubbles to pulsate in microgravity (Supplementary Video 6).

The rotation speed was determined using high-speed cameras that recorded the stirrer in motion under the same conditions as the experiments. The stirrer rotated with a period of 203 ms per rotation, resulting in an angular velocity *ω* ≈ 31 rad s^−1^. With a stirrer radius of 1.5 cm, the tangential velocity of the fluid was *v* ≈ 46.5 cm s^−1^.

#### Statistical analysis

Error propagation was evaluated for a sample size of *n* = 3. The standard deviation for three individual electrochemical experiments was calculated by computing the deviation of each value from their mean, then squaring these deviations to emphasize larger differences. The average of these squared deviations was calculated and the square root was taken to obtain the standard deviation, *σ*.

The shaded area around the mean lines of the same colour in Fig. 1a–c represent ±1*σ* to indicate the range where approximately 68% of the data points are expected to be found for normally distributed data populations. The error bars in the bar charts shown in Fig. 1d–f represent the s.e.m. The s.e.m. was calculated by dividing the standard deviation by the square root of the sample size (*n* = 3) to indicate how the sample mean varies from the true population mean. This provides a quantitative understanding of the precision of the experimental results.

### Prototype PEM device

#### Fabrication

A PEM model electrolyser cell with a platinum-coated PEM (Catalyst-Coated Nafion Membrane (CCM) N117, Ion Power; loading, 0.3 mg Pt cm^−^^2^; active area, 2.0 cm × 2.0 cm; overall area, 3.0 cm × 4.0 cm) was constructed according to Supplementary Fig. 9: the platinum-coated membrane was contacted with two platinum meshes (A-002250, Biologic) on both the anodic and cathodic sites. These meshes, originally 4 cm × 3 cm, were halved along the long side to create two 2 cm × 3 cm contact areas which were then pushed between two PEEK frames (2 cm × 1.9 cm) with holes for bubble removal. Additional platinum wires, 0.1 mm thick, were used to electrically contact the meshes from outside. The electrochemical cell was equipped with two cylindrical N52 neodymium permanent magnets (NdFeB, K&J Magnetics; length, 19.05 mm; diameter, 25.4 mm), each positioned at the anodic and cathodic site of the cell and aligned along the same axis with a separation distance of 22 mm. The resulting magnetic field is shown in Supplementary Fig. 11. The set-up included a PEEK electrolyser chamber with an acrylic glass cover for visual observation.

#### Electrochemical test

Chronopotentiometric experiments were carried out with the PEM electrolyser model at a constant current of 200 mA in ultrapure water (18.2 MΩ cm) while changes in the potential difference between the anode and cathode were measured over time *U*(*t*). To ensure valuable comparisons with terrestrial measurements, the electrolyser was operated for an additional 9.2 s after the capsule decelerated in the Bremen Drop Tower in a continuous experiment. Data were normalized by dividing the potential difference by the mean value of a selected time range during the microgravity phase. Specifically, for the microgravitation period, the normalization range was set to 5.5 s < *t* ≤ 8.5 s which reflects the time period between *U*(*t*) saturation and the onset of experiment deceleration. The maximum voltages recorded during *U*(*t*) measurements ranged from 5.3 V to 5.7 V. The deceleration shock caused a quick rise in potential that quickly returned to former values achieved in microgravity. The normalization was used to mitigate the influence of any potential offsets or differences in absolute values, allowing for a more direct observation and analysis of the relative changes in the potential difference.

#### Bubble velocity measurements

Video analysis of PEM microgravity experiments was conducted in MATLAB using the Image Processing Toolbox. A pixel-to-millimetre conversion was established using a known dimension, and exclusion boundaries were set to ignore bubbles that accumulated on the magnet casing. The algorithm captures centre locations and bubble radii at each frame, which were then sequenced into discrete bubble trajectories via proximity and velocity filters (Supplementary Fig. 10). The final collection was selected from this candidate pool based on gas bubbles that transit across the gap without becoming obscured by another bubble. In total, 41 hydrogen and 33 oxygen gas bubbles are shown in Supplementary Fig. 10a,b. Velocities were computed using a first-order finite-difference scheme, and data were smoothed with a moving-average filter over 0.3 s (Supplementary Fig. 10b). The average Reynolds numbers for hydrogen and oxygen bubbles were 0.88 (*σ* = 0.29) and 0.80 (*σ* = 0.29), respectively. The average number of frames included in the velocity analysis for each bubble was 116 (*σ* = 34.5). After nucleation, bubbles separated from the platinum mesh and were accelerated towards the magnets, where they accumulated in a stationary froth layer and sometimes coalesced into larger bubbles. Note that some bubbles approached the computed terminal velocity for a 1-mm bubble as shown in Supplementary Fig. 10b, which aligns with equation (9) in ref. ^25^ because terminal velocity scales with *R*^2^ in the Stokes regime. A similar bubble-analysis algorithm is described in detail in ref. ^25^. The maximum horizontal velocity reached by a tracked bubble was on average 2.77 mm s^−1^ (*σ* = 0.68) for hydrogen and 2.91 mm s^−1^ (*σ* = 0.65) for oxygen (Supplementary Fig. 11). The maximum velocity of 4.36 mm s^−1^ across all bubbles is slightly below the terminal velocity predicted by the quasistatic bubble balances in equations (9) and (10) in ref. ^26^ and computed in Supplementary Fig. 12 for creeping flows^26^. This is a consequence of the unsteady nature of the experiment, which prevents bubbles from reaching their terminal velocities before they are collected.

### Prototype MHD drive

#### Fabrication

A prototype for the MHD drive cell was constructed from three components: a main body, fabricated from PEEK that formed the central cavity for the platinum electrodes and the 1 M HClO_4_ (aq.) electrolyte plus a bottom and lid made from acrylic glass (Fig. 4a,b and Supplementary Fig. 12). Acrylic glass was chosen to ensure video recordings of the gas bubble flow during the experiment. The main body of the cell measured 21.1 mm in height and 80 mm in both width and depth. Around the body, a circular, thin cavity was carved out to accommodate eight N52 neodymium arc magnets supplied by K&J Magnetics. These eight segments formed a circular magnet with an internal diameter of 5.08 cm, a radial thickness of 3.175 mm and a height of 19.05 mm. To protect the magnets from corrosion during long-term electrolyte exposure, the cavity was encapsulated with the same black epoxy used for fabricating the other electrodes (UR 5528, Electrolube) and cured for 24 h.

To ensure air bubbles were not trapped inside the cell, a pump was used to evacuate the device prior to filling it with 26 ml electrolyte.

Two platinum rings were fabricated for the MHD drive system from a polycrystalline platinum foil (50 mm × 50 mm; thickness, 0.1 mm; purity grade, >99.99+%; polished surface; Goodfellow Cambridge) with an outer diameter of 47.1 mm and an inner diameter of 29.9 mm. The platinum foil rings were positioned in the cell using cylindrical fixtures to maintain equidistance between the rings (Fig. 4a,b). This positioning was crucial to ensure their proper placement in the middle of the magnetic field. Platinum wires encased in PTFE sleeves were used to establish an electrical contact with the platinum foil rings.

The magnetic field generated by the permanent magnet used in the MHD drive was computed as the superposition of the individual magnetic fields induced by *N* = 20 equivalent circular loops located at the top and bottom walls, which feature discontinuous tangent residual magnetization components. Each loop has a current *I*_loop_ = *M*_r_(*R*_magnet e_ − *R*_magnet i_)/*N*, with *R*_magnet__e_ and *R*_magnet__i_ being the outer and inner radii of the MHD drive, respectively, resulting in an analogous magnetic system with an analytical solution. The magnetic flux density field is represented in Fig. 4c. Further details on the virtual currents method can be found in ref. ^36^.

#### Angular velocity and bubble measurements

The angular velocity of the aqueous electrolyte inside the MHD drive was measured using bubbles as a visual reference. The angular velocity is approximately constant across all radii for a certain current intensity, making the electrolyte rotate as a solid cylinder except for localized regions such as the small liquid volume above the electrodes. Supplementary Fig. 14a depicts this behaviour for individual bubbles, while Fig. 5a shows an average of the observed angular velocity of 3–6 bubbles for each experiment. Steady-state angular velocities were computed by limiting bubble tracking to the last seconds of each drop or initiating the MHD drive operation slightly before the drop. For the chronopotentiometric measurements at 21, 42 and 63 mA cm^−2^, the angular velocity was lower, and the experiments started from a static state.

The bubble terminal velocity was obtained by manually tracking the radial position of 1- to 1.2-mm-diameter gas bubbles over time. The radial velocity of bubbles decreased with the radial position, but bubble–bubble interactions and the electrode wires shown in Fig. 4a,d induced transient flow dynamics that are not representative of the overall flow behaviour of the MHD drive. Isolated gas bubbles were selected at the 21, 42 and 63 mA cm^−2^ current levels shown in Supplementary Fig. 14b. For the gas bubble at a current density of 105 mA cm^−2^, however, the effect of transient interactions on the radial velocity was mitigated because the tracking was carried out along a wide range of angular positions. The bubble interacts with the wire for radial positions between 1.3 and 1 cm, where the radial velocity notably fluctuates.

#### Electrochemical tests

Chronopotentiometric experiments were carried out for 9.2 s at 1,000 mA in a two-electrode set-up, measuring the potential difference between the two platinum electrodes inside the MHD drive cell. All data were recorded by a capsule computer (National Instruments). The experimental sequence was adapted from previous electrochemical experiments with minor adjustments.

Microgravity experiments started with the trigger of the microgravity switch. Unlike the angular velocity measurement, and to capture the initial transient behaviour, the electrochemical characterization was performed by starting the rotation of the system at the beginning of the drop. After the capsule landed again in the deceleration container, a brief pause of 10 s ensured capsule stabilization before another experiment with the same CP settings was carried out terrestrially for an additional 9.2 s. This repetition under terrestrial conditions served as a reference point for further analyses (Fig. 5c).

## Online content

Any methods, additional references, Nature Portfolio reporting summaries, source data, extended data, supplementary information, acknowledgements, peer review information; details of author contributions and competing interests; and statements of data and code availability are available at 10.1038/s41557-025-01890-0.

## Supplementary information

Supplementary Information Supplementary Figs. 1–15.

Supplementary Video 1 HER on Pt foil with and without magnet in *g* and μ*g*.

Supplementary Video 2 HER on Pt mesh with and without magnet in *g* and μ*g*.

Supplementary Video 3 OER on IrOx with and without magnet in *g* and μ*g*.

Supplementary Video 4 HER on photoelectrodes with and without magnet.

Supplementary Video 5 HER on Pt foil with and without magnet at low current density.

Supplementary Video 6 HER during stirring/absence of stirring.

Supplementary Video 7 PEM model in μ*g* with magnets.

Supplementary Video 8 MHD drive in μ*g*.

Supplementary Video 9 MHD drive in *g*.

## Source data

Source Data Fig. 1Current measurements normalized by electrode surface area to yield current density (mA cm^−2^). Data correspond to platinum foil (a), platinum mesh (b) and IrO_*x*_ (c) on gold electrodes under different magnetic field and gravity conditions (terrestrial *g*_0_, 1*g*; microgravity μ*g*, 10^−6^*g*; with (mag)/without magnet (nomag)). Includes time-resolved current density, current at 8 s (${{\rm{J}}}_{{8}_{{\rm{s}}}}$), and rate of change (d*J*/d*t*). Used to generate all panels a–f in Fig. 1.

Source Data Fig. 3 Normalized current density data of conducted proton-exchange membrane electrolyser experiments. Used to generate panel b in Fig. 3.

Source Data Fig. 5 Angular and radial velocity measurements under varying current densities in *g*_0_ (1*g*) and μ*g* (10^−6^*g*) environments. Includes angular velocity versus current density (a), radial gas bubble velocity at different current density values (b) and constant potential measurements at 105 mA cm^−2^ in 0*g* versus 1*g* (c). Data correspond to MHD drive performance under terrestrial (*g*_0_) and microgravity (μ*g*) conditions. Presented as mean ± 2 s.d. from three independent measurements. Used to generate all panels a–c in Fig. 2.

## Data Availability

All raw data for the paper figures and the supplementary figures are available on figshare at https://figshare.com/s/3102e2089938f54edece (ref. ^39^). Source data are provided with this paper.
