# Supplementary material for: Magnetically induced convection enhances water electrolysis in microgravity
Source: Nat Chem. 2025 Aug 18;17(11):1673–9. doi: 10.1038/s41557-025-01890-0 (PMC12580345; doi:10.1038/s41557-025-01890-0)
Supplement: Supplementary file 1 — Supplementary Figs. 1–15. [file 41557_2025_1890_MOESM1_ESM.pdf]

nature chemistry

Supplementary information

https://doi.org/10.1038/s41557-025-01890-0

# Magnetically induced convection enhances water electrolysis in microgravity

In the format provided by the authors and unedited

## Table of Contents


| | Page |
|---|---|
| **Supplementary Fig. 1.** Overview of the two-compartment (photo)electrochemical cell with two independent three-electrode setups employed in *μg* and $g_0$ experiments. | 2 |
| **Supplementary Fig. 2.** Magnetic environment in the experimental setup in the plane defined by W.E. and the C.E. | 3 |
| **Supplementary Fig. 3**. CA hydrogen evolution measurements in *μg* using p-type InP photoelectrodes with a nanostructured Rh electrocatalyst coating in the presence and absence of a magnetic field under illumination at 89 mW $cm^{-2}$ (W-I lamp). | 4 |
| **Supplementary Fig. 4.** CA hydrogen evolution measurements using polycrystalline Pt foil electrodes in the presence of a magnetic field in $g_0$ and *μg* environments. | 5 |
| **Supplementary Fig. 5.** Schematic illustration of the stirrer attached to the electrochemical cell. | 6 |
| **Supplementary Fig. 6.** CA hydrogen evolution measurements using polycrystalline Pt electrodes in the presence and absence of a magnetic field as well as mechanical stirring in $g_0$ and *μg* environments. | 7 |
| **Supplementary Fig. 7.** Time series of hydrogen gas bubble evolution during CA measurements on Pt foil electrodes in the presence and absence of a magnetic field in *μg* and $g_0$ environments. | 8 |
| **Supplementary Fig. 8.** Time series of oxygen gas bubble evolution during CA measurements on $IrO_x$ electrodes in the presence and absence of a magnetic field in *μg* and $g_0$ environments. | 9 |
| **Supplementary Fig. 9.** PEM electrolyser model construction. | 10 |
| **Supplementary Fig. 10.** Raw cell voltage measurements of the PEM electrolyser model device during *μg* and consecutive $g_0$ experiments. | 11 |
| **Supplementary Fig. 11.** Magnetic environment of the PEM electrolyser prototype. | 12 |
| **Supplementary Fig. 12.** Scheme of a proof-of-concept cylindrical MHD drive electrolytic cell architecture exploiting the Lorentz force to induce vortical phase separation in *μg.* | 13 |
| **Supplementary Fig. 13.** Performance of the cylindrical MHD electrolytic cell prototype with and without external magnets in $g_0$. | 14 |
| **Supplementary Fig. 14.** Gas bubble dynamics within the MHD Drive prototype in steady-state conditions for different current densities in *μg.* | 15 |
| **Supplementary Fig. 15.** Trajectories and velocities of tracked bubbles acquired via video analysis in the model PEM electrolyser cell in *μg.* | 16 |

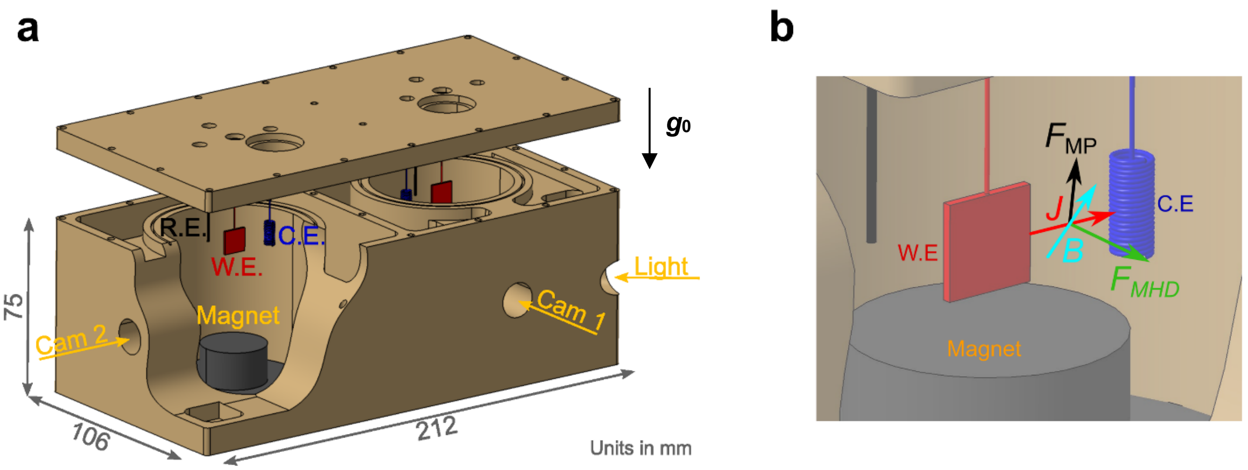


**Supplementary Fig. 1. Overview of the two-compartment (photo)electrochemical cell with two independent three-electrode setups employed in $\mu g$ and $g_0$ experiments. a** CAD model with dimensions in mm. The gravity vector orientation during terrestrial experiments is indicated. In microgravity, this also indicates the direction of flight. Further details on the setup can be found in the manuscript. **b** Detailed view of the electrochemical cell cavity showing the magnetic flux density field (blue), the current density field (red), and the resulting Lorentz force on the conductive liquid (green, $F_{MHD}$), together with the polarization force on the liquid (black, $F_{MP}$) for the oxygen evolution reaction at the working electrode (W.E.). C.E. is the counter electrode and R.E. the reference electrode. "Cam 1" and "Cam 2" indicate the camera positions and "Light" indicates the window through which setup and sample illumination occurred.

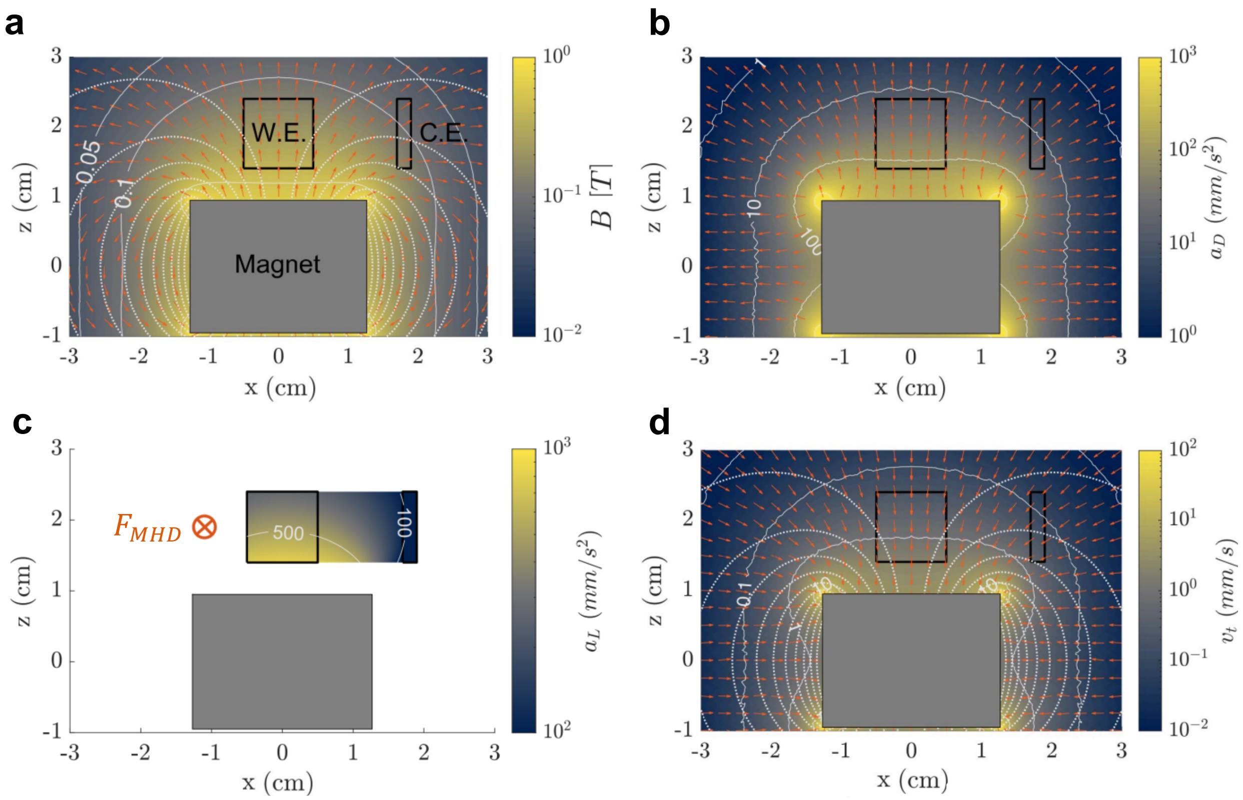


**Supplementary Fig. 2. Magnetic environment in the experimental setup in the plane defined by W.E. and the C.E.. a** Magnetic flux density. **b** Diamagnetic acceleration on the liquid electrolyte assuming the magnetic susceptibility of ultrapure water (equation (2). **c** Approximate Lorentz-force acceleration at a current density of 200 mA $cm^{-2}$ based on equation (1) and the magnetic field map, assuming uniform current lines between the electrodes. **d** Diamagnetic terminal velocity for a 1 mm diameter bubble using equation (10) in ref. 26. W.E. and C.E. are both scetched after Supplementary Fig. 1 and represent e.g., the planar Pt foil and the $IrO_x$ electrode. The volume forces in equations 1 and 2 can be converted to accelerations through $a = F/\rho$, with $\rho$ being the density of the electrolyte.

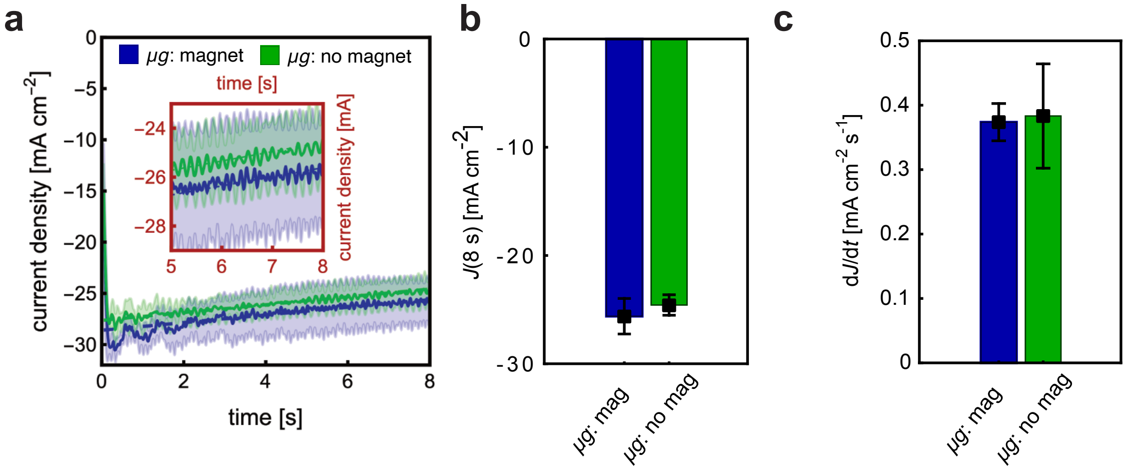


**Supplementary Fig. 3. CA hydrogen evolution measurements in *μg* using p-type InP photoelectrodes with a nanostructured Rh electrocatalyst coating in the presence and absence of a magnetic field under illumination at 89 mW cm$^{-2}$ (W-I lamp). a** Measurements in terrestrial and microgravity environments were carried out at -0.09 V *vs.* NHE for a minimum of 8 s each in 1M $HClO_4$(aq) with the addition of 1% (v/v) 2-propanol. Linear regression analyses were carried out for t > 3 s. **b** shows the current density in these two environments after 8 s. The decrease during the experiment was calculated as a function of time (*dJ/dt*), **c**. The error bars in **b** and **c** were calculated based on three independent measurements.

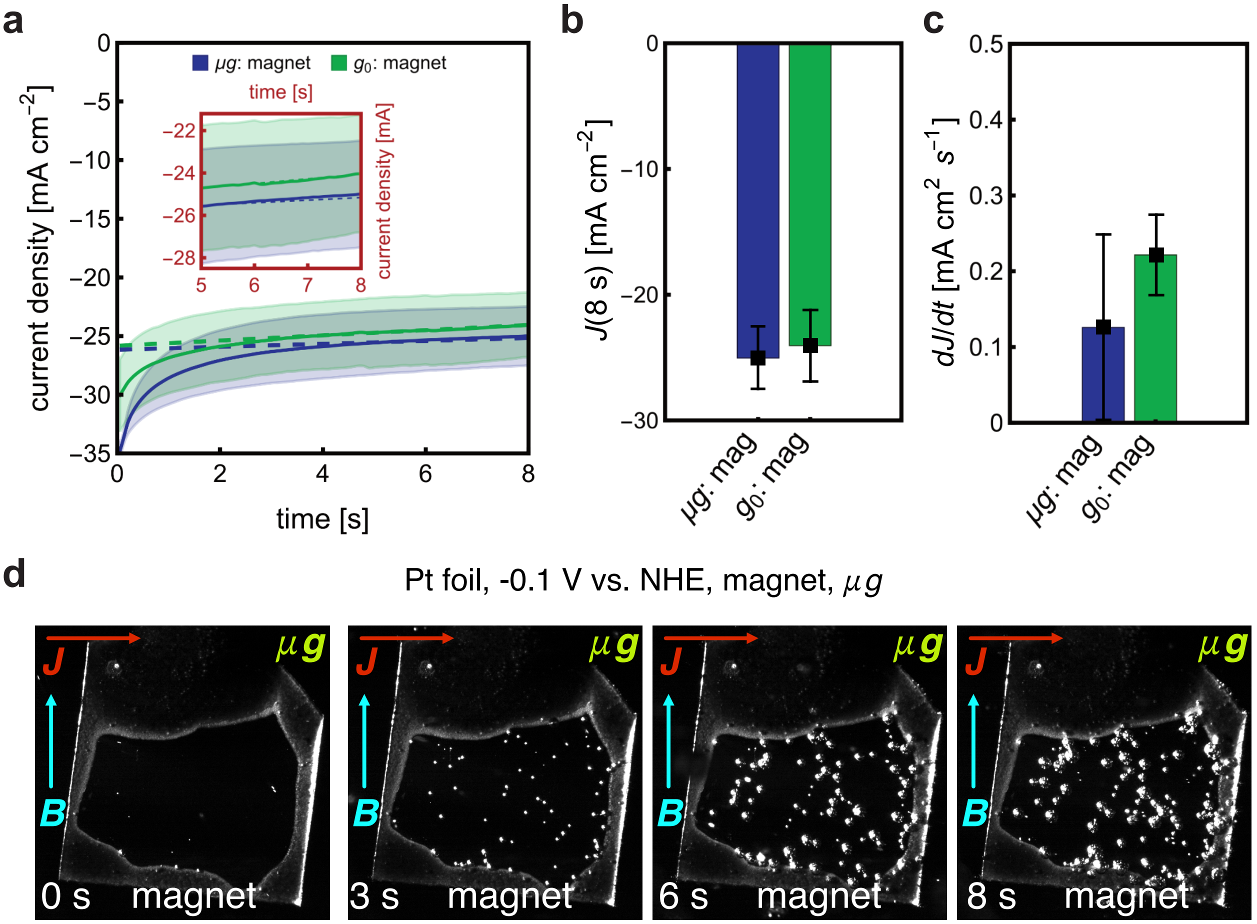


**Supplementary Fig. 4. CA hydrogen evolution measurements using polycrystalline Pt foil electrodes in the presence of a magnetic field in $g_0$ and $\mu g$ environments. a** Measurements were carried out at -0.1 V *vs.* NHE for 8 s each. Linear regression analyses were performed for t > 3 s in **a**. **b** shows the slope of the current density decrease over time (*dJ/dt*) and **c** shows the current density at 8 s. The error bars were calculated based on three independent measurements. **d** Time series of the hydrogen gas bubble evolution during the CA measurements on Pt foil electrodes in the presence of a magnetic field in microgravity environment. Snapshots were extracted from high frame rate footage and were taken at four specific time intervals, commencing just ~100 ms after the onset of acceleration within the Bremen Drop Tower until 8 s after beginning of the microgravity phase.

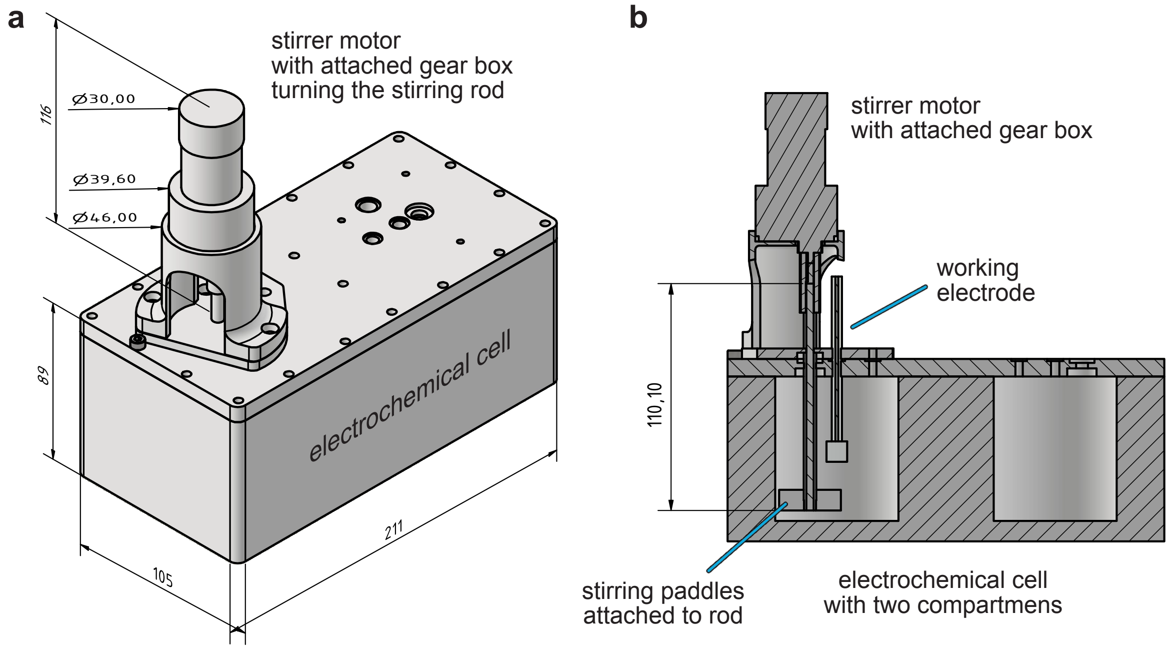


**Supplementary Fig. 5. Schematic illustration of the stirrer attached to the electrochemical cell. a** shows an outside view and **b** the stirrer position inside the cell. Size dimensions are in mm.

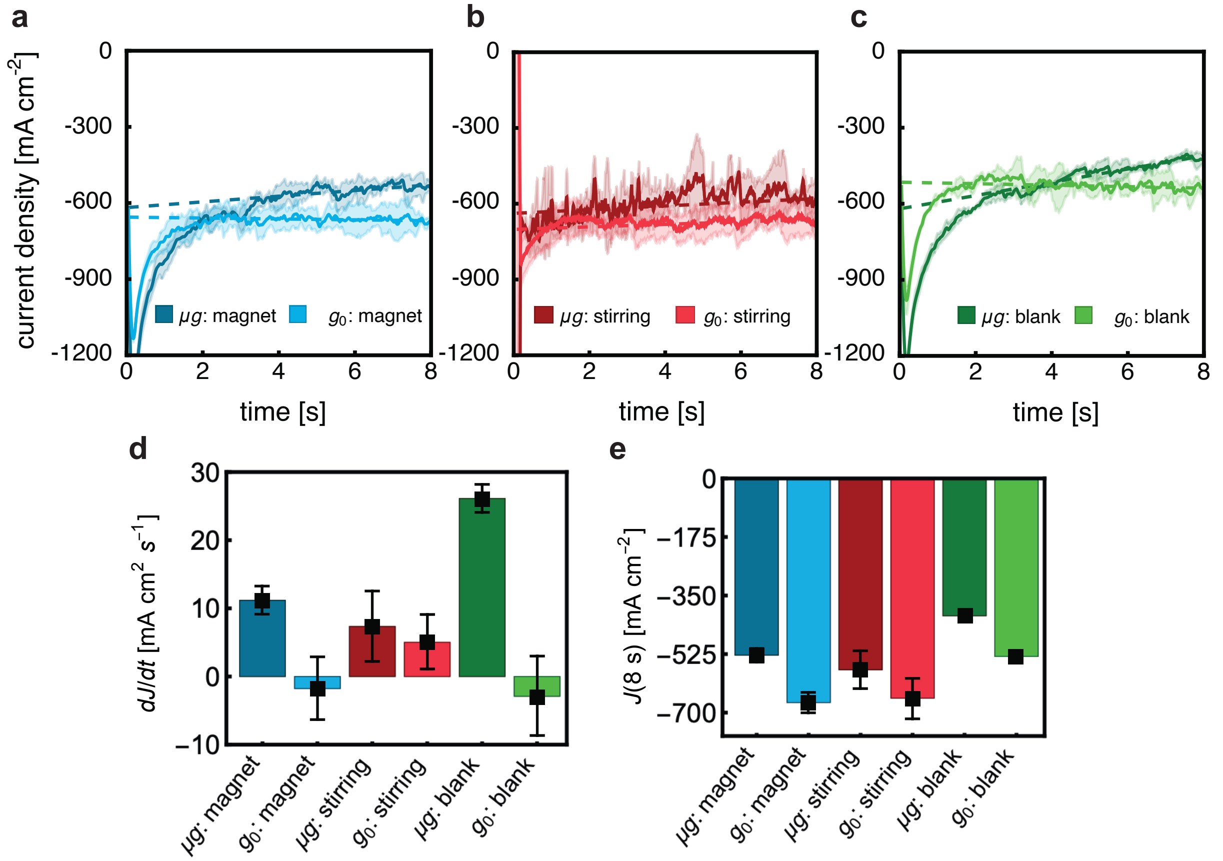


**Supplementary Fig. 6. CA hydrogen evolution measurements using polycrystalline Pt electrodes in the presence and absence of a magnetic field as well as mechanical stirring in $g_0$ and *μg* environments.** Measurements were carried out in the HER (**a** - **c**) regions for 8 s each. **a** shows the HER CA characteristics in the presence of a magnetic field in $g_0$ and *μg*, **b** shows the HER CA characteristics in the presence of mechanical stirring of the electrolyte at -1.6 V *vs.* NHE in $g_0$ and μ*g* and **c** shows the HER CA characteristics in the absence of mechanical stirring as well as a magnetic field in $g_0$ and *μg*. Linear regression analyses were performed for t > 3 s in **a** - **c**. **d** and **e** show the slopes of the current density decrease over time (*dJ/dt*) and the current density at 8 s for all six measurements. Data are presented as mean values ± one standard deviation from three independent measurements (n = 3).

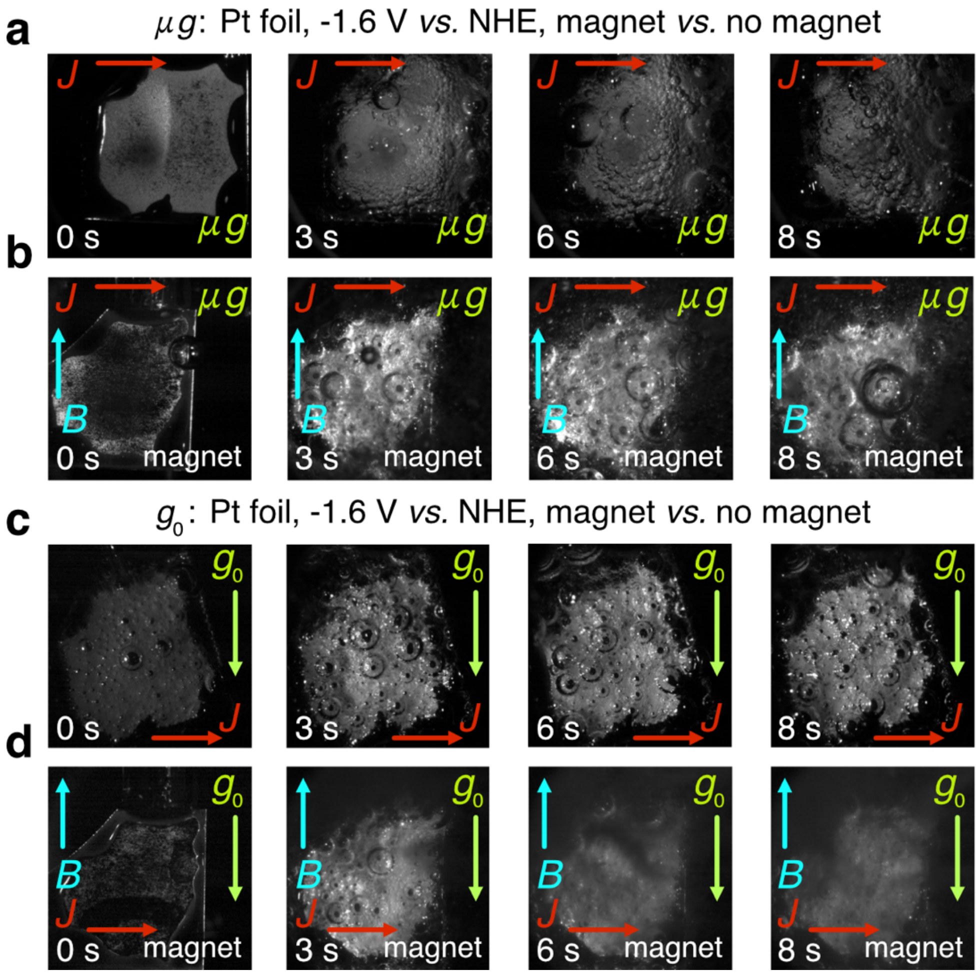


**Supplementary Fig. 7. Time series of hydrogen gas bubble evolution during CA measurements on Pt foil electrodes in the presence and absence of a magnetic field in *μg* and *$g_0$* environments.** The voltage applied at the Pt foil electode was -1.6 V *vs.* NHE. Snapshots were extracted from high frame rate footage and were taken at four specific time intervals, commencing just ~100 ms after the onset of acceleration within the Bremen Drop Tower until 8 s after beginning of the microgravity phase. **a** Without a magnetic field, hydrogen gas bubbles coalesce and form a froth layer on the Pt electrode in microgravity. **b** The magnet positioned at the bottom of the electrode attracts hydrogen gas bubbles through the magnetic polarization of the electrolyte. This induces convection and facilitates gas bubble removal by introducing an artificial buoyancy effect, causing an overall thinner foam layer on the electrode. **c** For comparison, terrestrial experiments were conducted using the same electrode without and with a magnet (**d**). All videos are available as Supplementary Video 1.

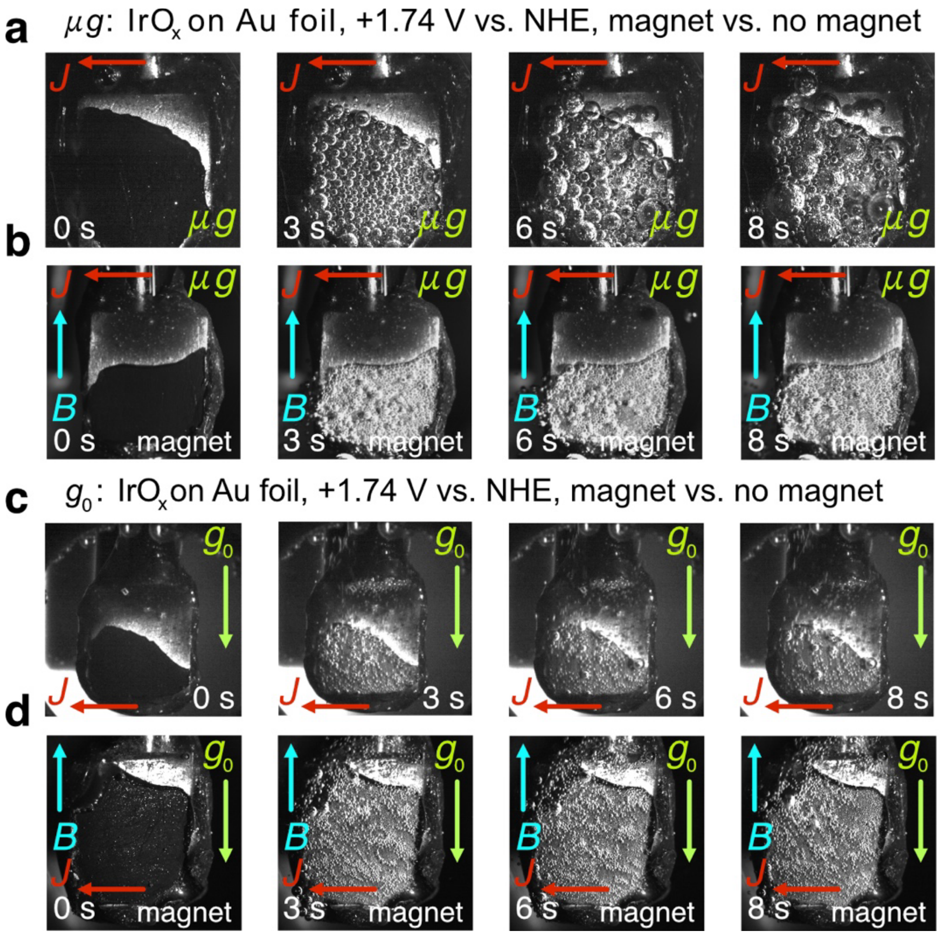


**Supplementary Fig. 8. Time series of oxygen gas bubble evolution during CA measurements on $IrO_x$ electrodes in the presence and absence of a magnetic field in *μg* and *$g_0$* environments.** The voltage applied at the $IrO_x$ electrode was +1.74 V *vs.* NHE. Snapshots were extracted from high frame rate footage and were taken at four specific time intervals, commencing ~100 ms after the onset of acceleration within the Bremen Drop Tower until 8 s after beginning of the microgravity phase. **a** Without a magnetic field, oxygen gas bubbles coalesce and form a froth layer on the electrode in microgravity. **b** The magnet positioned at the bottom of the $IrO_x$ electrode attracts oxygen gas bubbles through the magnetic polarization of the electrolyte. This induces convection and facilitates gas bubble removal by introducing an artificial buoyancy effect, causing an overall thinner foam layer on the electrode. **c** For comparison, terrestrial experiments were conducted using the same electrode without and with a magnet (**d**). All videos are available as Supplementary Video 3.

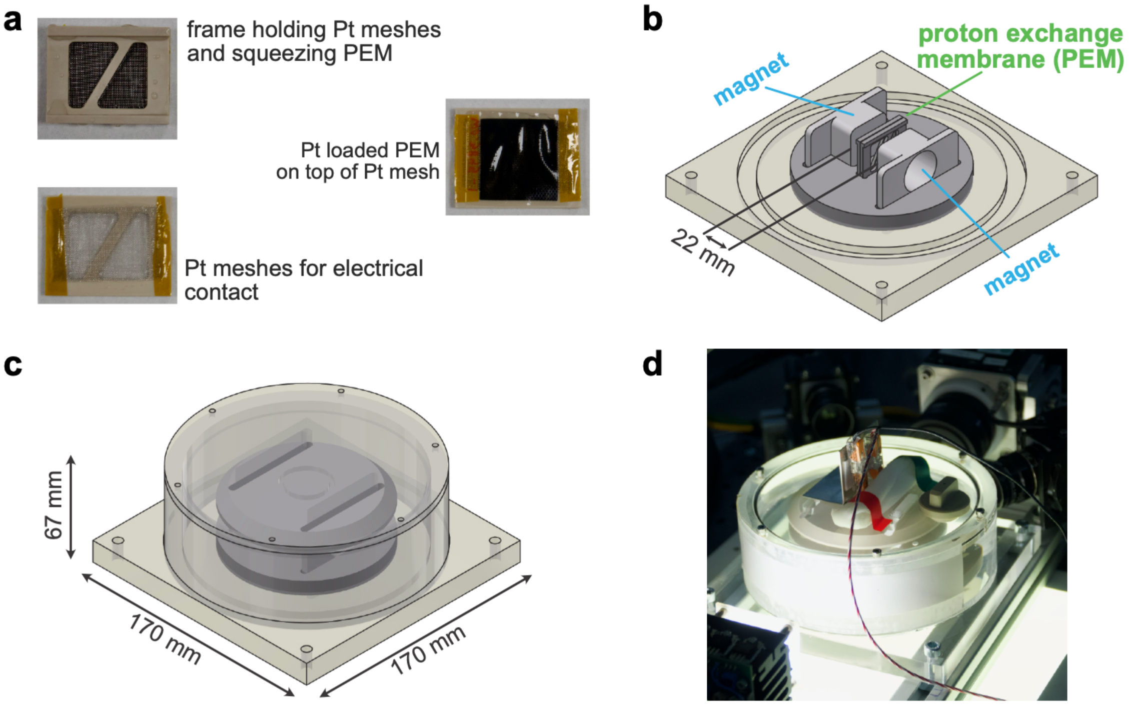


**Supplementary Fig. 9. PEM electrolyser model construction.** **a** Step-by-step construction of the model electrolyser. The Pt-coated PEM was contacted with a Pt mesh on both, the anodic and cathodic site. Two PEEK frames on each site squeezed the three elements together. **b** The PEM was then sliced into a holder and two neodymium permanent magnets were located opposite of the anodic and cathodic site, respectively. They were protected from the electrolyte by a PTFE case. **c** The PEM model was placed into a cylindrical outer shell made of transparent acrylic glass for video capture that also served as an electrolyte basin. **d** Image of the final cell set-up in the drop capsule with ambient background lighting and high-speed video cameras.

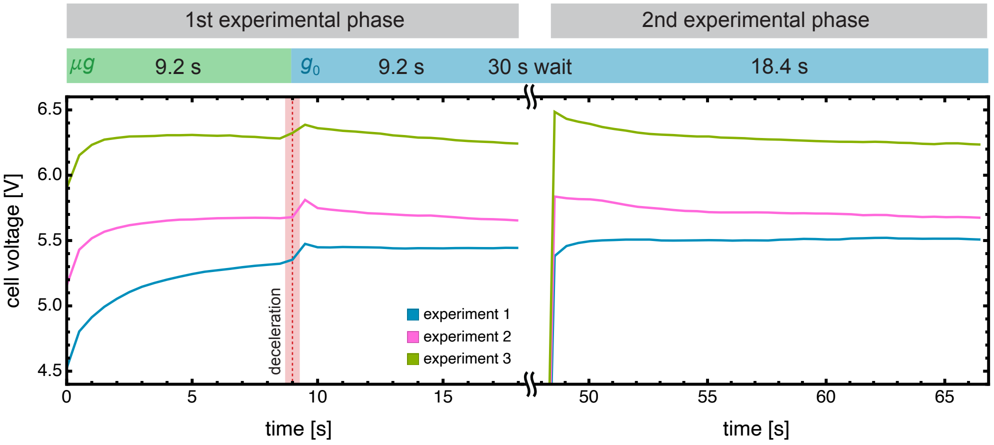


**Supplementary Fig. 10. Raw cell voltage measurements of the PEM electrolyser model device during *μg* and consecutive $g_0$ experiments.** Shown are the cell voltage [V] *vs.* time [s] characteristics for three independent experiments. Each experiment was carried out in two phases as illustrated. Phase one occurred during the transition between microgravity (*μg*, 0 to 9.3 s) and terrestrial gravitation ($g_0$, 9.3 to 18.4 s), showing the initial voltage response in microgravity followed by a voltage spike during decelaration (marked by the vertical line in red). After a 30 s wait period (break in x-axis), the second experimental phase shows the voltage behaviour during terrestrial operation.

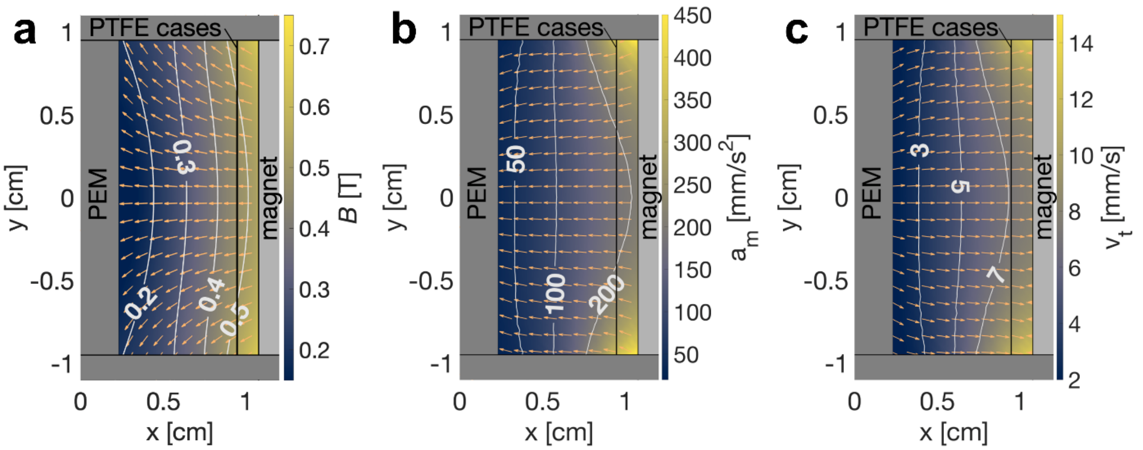


**Supplementary Fig. 11. Magnetic environment of the PEM electrolyser prototype. a** Magnetic flux density in the region of bubble travel. **b** Diamagnetic acceleration field imparted to the liquid by the magnets (equation (2)). **c** Terminal velocity of a 1 mm diameter gas bubble in water using equation (10) in ref. 26. Only the anodic site of the symmetric PEM - magnets assembly is shown.

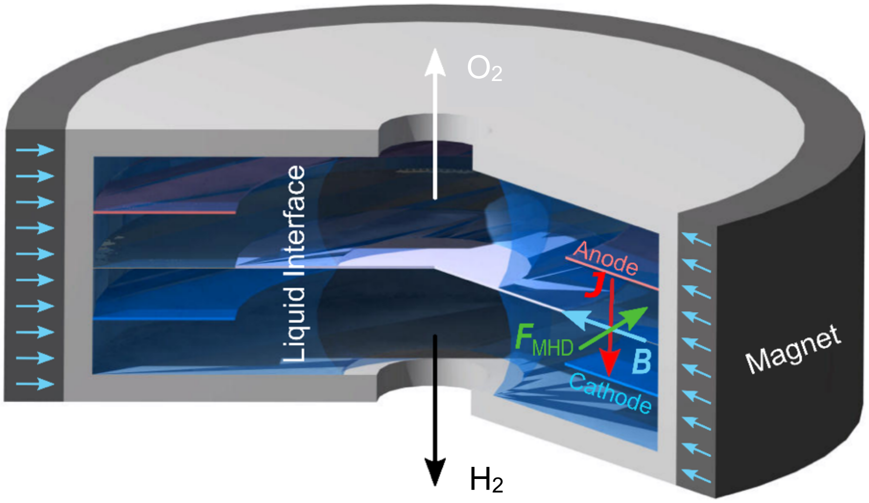


**Supplementary Fig. 12. Scheme of a proof-of-concept cylindrical MHD drive electrolytic cell architecture exploiting the Lorentz force to induce vortical phase separation in *µg*.** The combination of a radial magnetic field and the electrical current density between anode and cathode generates a Lorentz force on the liquid that induces a vortical flow. Phase separation is initiated due to the centrifugal acceleration and the density difference between liquid and gas phases. Note: the membrane indicated in the middle of the drive was not present in the prototype under study due to the short experiment time in microgravity.

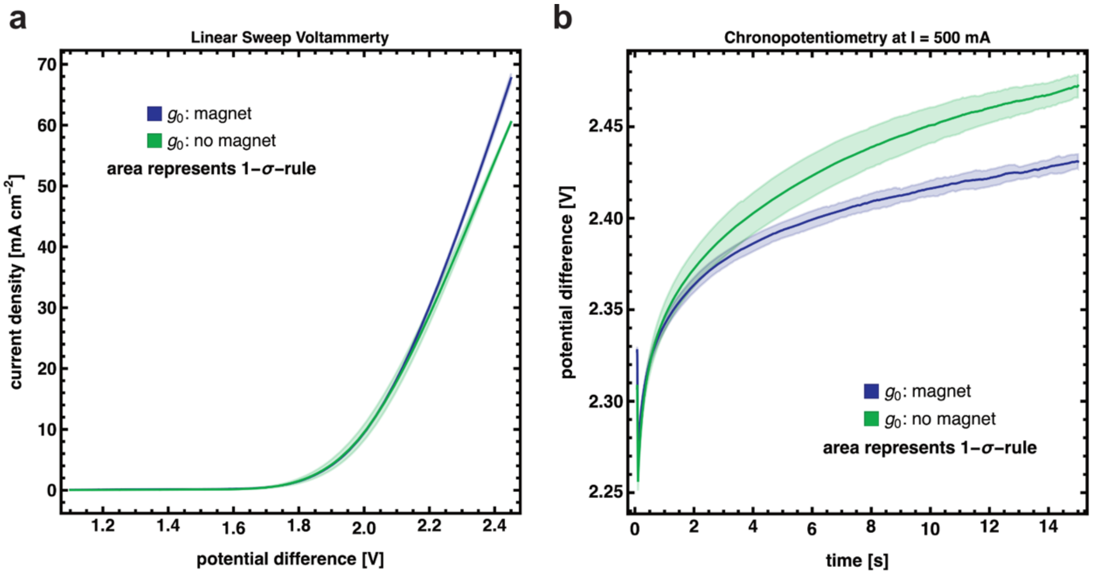


**Supplementary Fig. 13. Performance of the cylindrical MHD electrolytic cell prototype with and without external magnets in $g_0$. a** Linear sweep voltammetry. **b** Chronopotentiometry at I = 500 mA (corresponding to 52 mA cm$^{-2}$).

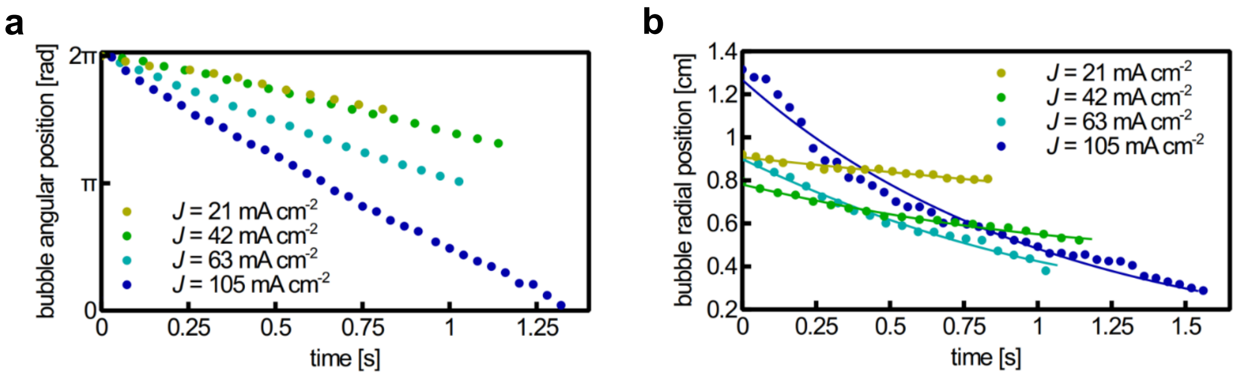


**Supplementary Fig. 14. Gas bubble dynamics within the MHD Drive prototype in steady-state conditions for different current densities in *μg*. a** Angular bubble positions as a function of time. **b** Radial bubble positions as a function of time. The points show the experimental data while the fitted lines are used to estimate the radial velocity of the bubbles.

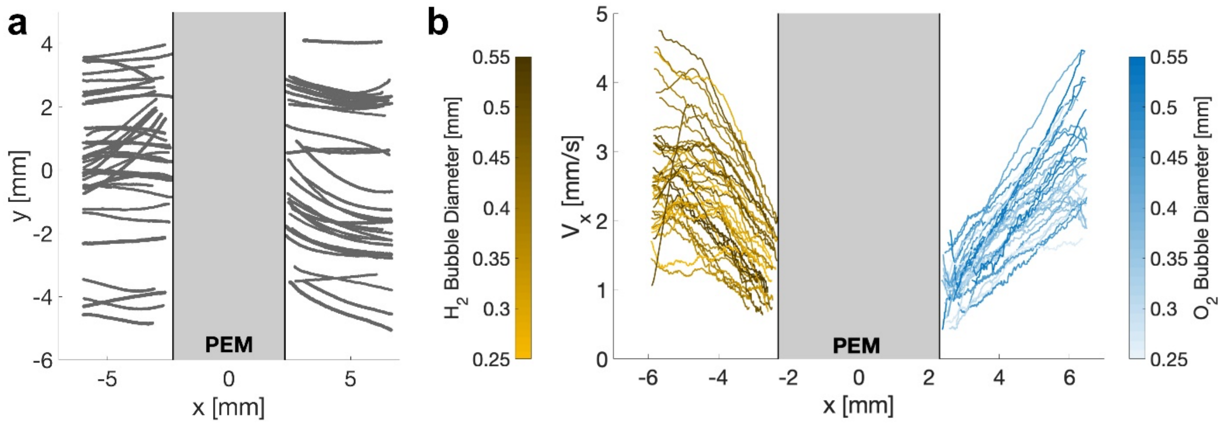


**Supplementary Fig. 15. Trajectories and velocities of tracked bubbles acquired via video analysis in the model PEM electrolyser cell in *µg*. a** Each line depicts extracted center location from a series of frames of a single bubble. **b** Absolute values of bubble horizontal velocities are shown as a function of x-location.
